# Targeting SxIP-EB1 interaction: An integrated approach to the discovery of small molecule modulators of dynamic binding sites

**DOI:** 10.1038/s41598-017-15502-6

**Published:** 2017-11-14

**Authors:** T. B. Almeida, A. J. Carnell, I. L. Barsukov, N. G. Berry

**Affiliations:** 1 University of Liverpool, Department of Chemistry, Liverpool, L69 7ZD United Kingdom; 2 University of Liverpool, Institute of Integrative Biology, Liverpool, L69 7ZB United Kingdom

## Abstract

End binding protein 1 (EB1) is a key element in the complex network of protein-protein interactions at microtubule (MT) growing ends, which has a fundamental role in MT polymerisation. EB1 is an important protein target as it is involved in regulating MT dynamic behaviour, and has been associated with several disease states, such as cancer and neuronal diseases. Diverse EB1 binding partners are recognised through a conserved four amino acid motif, (serine-X-isoleucine-proline) which exists within an intrinsically disordered region. Here we report the use of a multidisciplinary computational and experimental approach for the discovery of the first small molecule scaffold which targets the EB1 recruiting domain. This approach includes virtual screening (structure- and ligand-based design) and multiparameter compound selection. Subsequent studies on the selected compounds enabled the elucidation of the NMR structures of the C-terminal domain of EB1 in the free form and complexed with a small molecule. These structures show that the binding site is not preformed in solution, and ligand binding is fundamental for the binding site formation. This work is a successful demonstration of the combination of modelling and experimental methods to enable the discovery of compounds which bind to these challenging systems.

## Introduction

**Figure 1:**
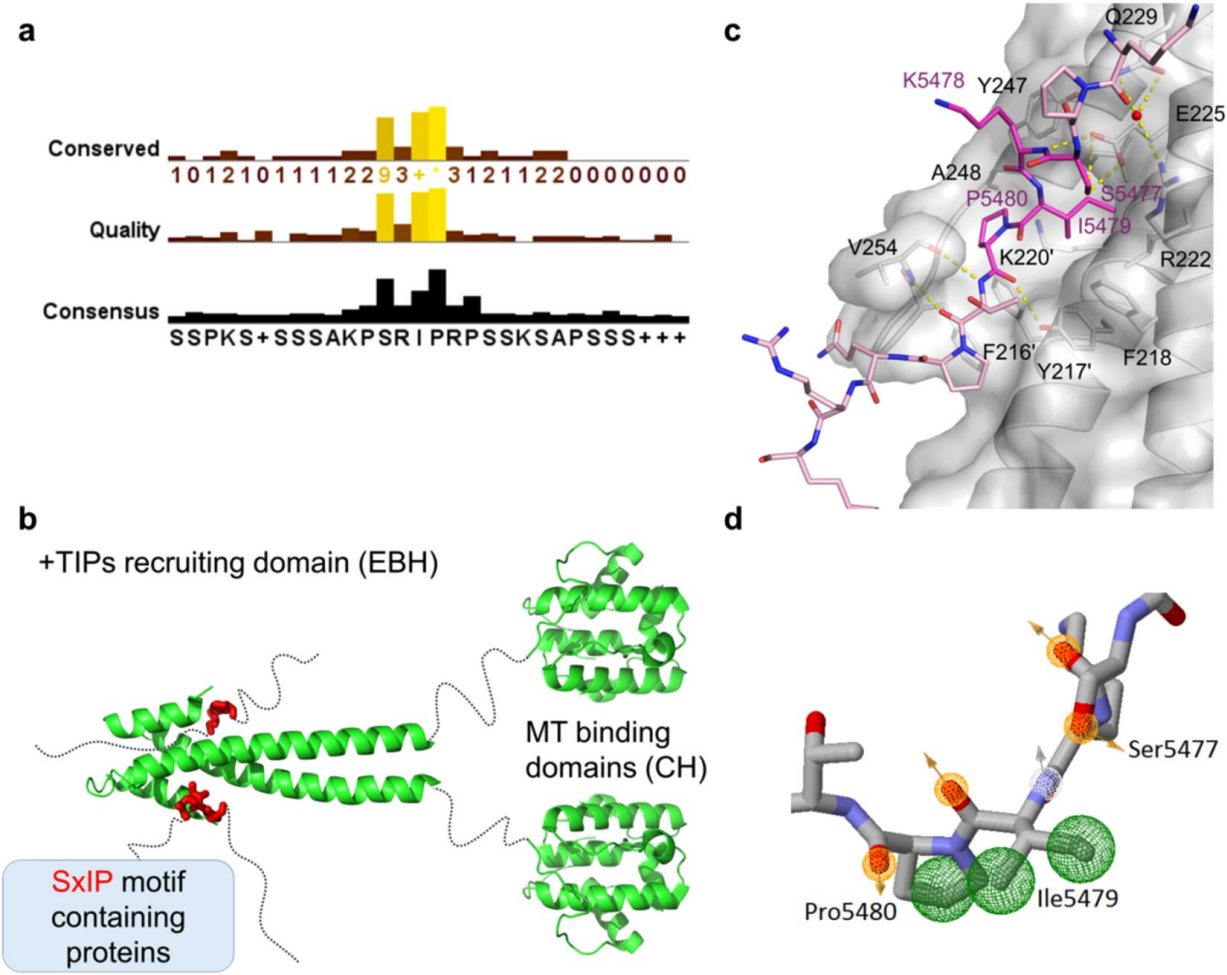
(**a**) Conservation analysis for known SxIP proteins based on a 30 residue sequence encompassing the identified SxIP motif. Figure was made using JalView 2.8.2. (**b**) Model for the mechanism of the recruitment of the SxIP motif containing proteins by EB1. Both EB1 domains are shown in green – the microtubule binding domain – calponin homology (CH) domain and the + TIPs recruitment domain – EBH domain. The SxIP motif is shown as red sticks within a disordered region represented by a dashed line. There are more than 42 proteins known to bind to EB1 via this conserved motif. (**c**) Representation of the SxIP crystallographic binding mode as shown by Honnappa *et al*.^5^ SxIP containing peptide is shown as light pink sticks, and the SxIP motif is highlighted as bright pink sticks. EB1 is shown as cartoon and surface representations with important residues shown as sticks. (**d**) Representation of the pharmacophore points found for Ser5477, Ile5479 and Pro5480 of the MACFp1 peptide. All pharmacophore features are shown as spheres, with the hydrogen bonding acceptors showed as orange mesh, hydrogen bonding acceptors in white mesh and hydrophobic as green mesh. Orange and grey arrows indicate the direction of the hydrogen bond donor/acceptor, respectively.

There are a diverse group of proteins known as plus-end tracking proteins (+TIPs), that regulate MT behaviour and interactions between MTs and other intracellular structures^1–3^. EB1, a member of the end binding (EB) family, has been shown to bind directly to MTs and to a wide range of +TIPs and cytoskeletal proteins, thus recruiting them to the plus-ends. EB1 consists of two domains linked through a flexible linker - the Calponin homology (CH) domain that binds directly to the microtubules and the End Binding homology (EBH) domain, the recruiting domain (Fig. 1b). Mutagenesis analysis revealed that these +TIPs bind to EB1 via either a conserved dipeptide Ile-Pro (IP motif) or a small four residue motif Ser-x-Ile-Pro – the SxIP motif^4,5^. The proteins in this diverse group, referred to as the SxIP proteins, have a variety of structures and functions. This conserved motif is common to at least 42 proteins, showing little variability – Fig. 1 panel a and Supplementary Fig. S1. The serine can only be replaced by threonine, the x residue is normally a positively charged residue (arginine or lysine), isoleucine can only be replaced by the hydrophobic residue leucine with loss of affinity and the proline is fully conserved^6^. One important common feature of the group is that the conserved SxIP motif is located in intrinsically disordered regions (IDRs) (Fig. 1b), enriched in basic, serine and proline residues.

Many of the SxIP proteins are linked to various diseases. For example, the mitotic centromere-associated kinesin (MCAK) regulates microtubule dynamics in depolymerisation of microtubules by removing tubulin subunits from the polymer end. This is important for ensuring the correct segregation of chromosomes in mitosis and for avoiding chromosome instability, a process common to many solid tumours^7^. The microtubule-actin crosslinking factor (MACF) is an integrator of MT-actin dynamics and implicated in breast carcinoma cell motility^8^ and the adenomatous polyposis coli (APC) protein is associated with colon carcinoma^1,9^. Thus, targeting the EB1-SxIP interaction with small molecules has a high therapeutic potential.

Inhibiting protein-protein interactions (PPIs) with small molecules is recognized as a major challenge in drug discovery^10^. The intrinsically disordered and dynamic regions present both in EB1 and its binding partners add to the complexity of the system. The crystallographic structures published to date do not show the C-terminal residues of EB1 indicating a highly dynamic region. The same is true for the 30 residue peptide derived from the C-terminal region of human MACF2 (residues 5468–5497), where the electron density only permits the observation of between 5 to a maximum of 11 residues^5^. Dynamic regions in protein-protein interfaces are common, but add extra difficulty for finding PPI modulators. Nevertheless, and despite these difficulties, a well-defined binding pocket for the SxIP motif detected in the crystal structure of the EB1 complex with MACF^5^ provides a good starting point in the search for potential modulators.

We report herein the first molecular scaffold to target the SxIP recruiting site of EB1. In a targeted approach based on a combination of pharmacophore searching, docking and multiparameter compound selection we have identified a range of small molecule candidates designed to modulate the EB1:SxIP interaction. We have examined the interactions of several compounds with EB1’s recruiting domain and solved the structure of the complex of EB1 with one of them by NMR. Our structural information reveals that the SxIP binding pocket incorporates several dynamic side-chains that become immobilised upon complex formation. Our approach provides an alternative strategy to high throughput screening (HTS) or fragment based design and should be particularly useful when targeting dynamic PPIs involving disordered regions.

## Results

The C-terminal domain of EB1 (EB1c) contains a unique EB-homology domain (EBH) and a disordered C-terminal tail^11^. This domain is responsible for the formation of a homodimer, and folds into a very stable coiled coil. A crystal structure of EB1 bound to a SxIP containing peptide^5^ (PDB code 3GJO) shows that SxIP motif binds to a well-defined hydrophobic cavity at the end of the EBH domain; part of the binding pocked is formed by the C-terminal region that folds over the bound peptide (Fig. 1c). The most important contacts involve Ser5477, Ile5479 and Pro5480, positions 1, 3 and 4 of the SxIP motif. Ser5477 forms an extensive network of hydrogen bonds with highly conserved Arg222, Glu225, Gln229 and Tyr247 via a coordinated water molecule. Lys5478 is within a salt bridge distance (4 Å) to Asp257; however, the electron density is poorly defined for both side chains, indicative of a dynamic region. Importantly, Ile5479 and Pro5480 are buried within a hydrophobic cavity defined by Leu221, Leu246, Phe216’, Tyr217’, Phe218, Lys220’, Arg222, Glu225, Tyr247, and Ala248 (where ‘ refers to the homodimeric partner) (Fig. 1c)^5^. We have exploited this detailed structural information via virtual screening and docking approaches to identify hit ligands.

### Virtual screening

**Figure 2:**
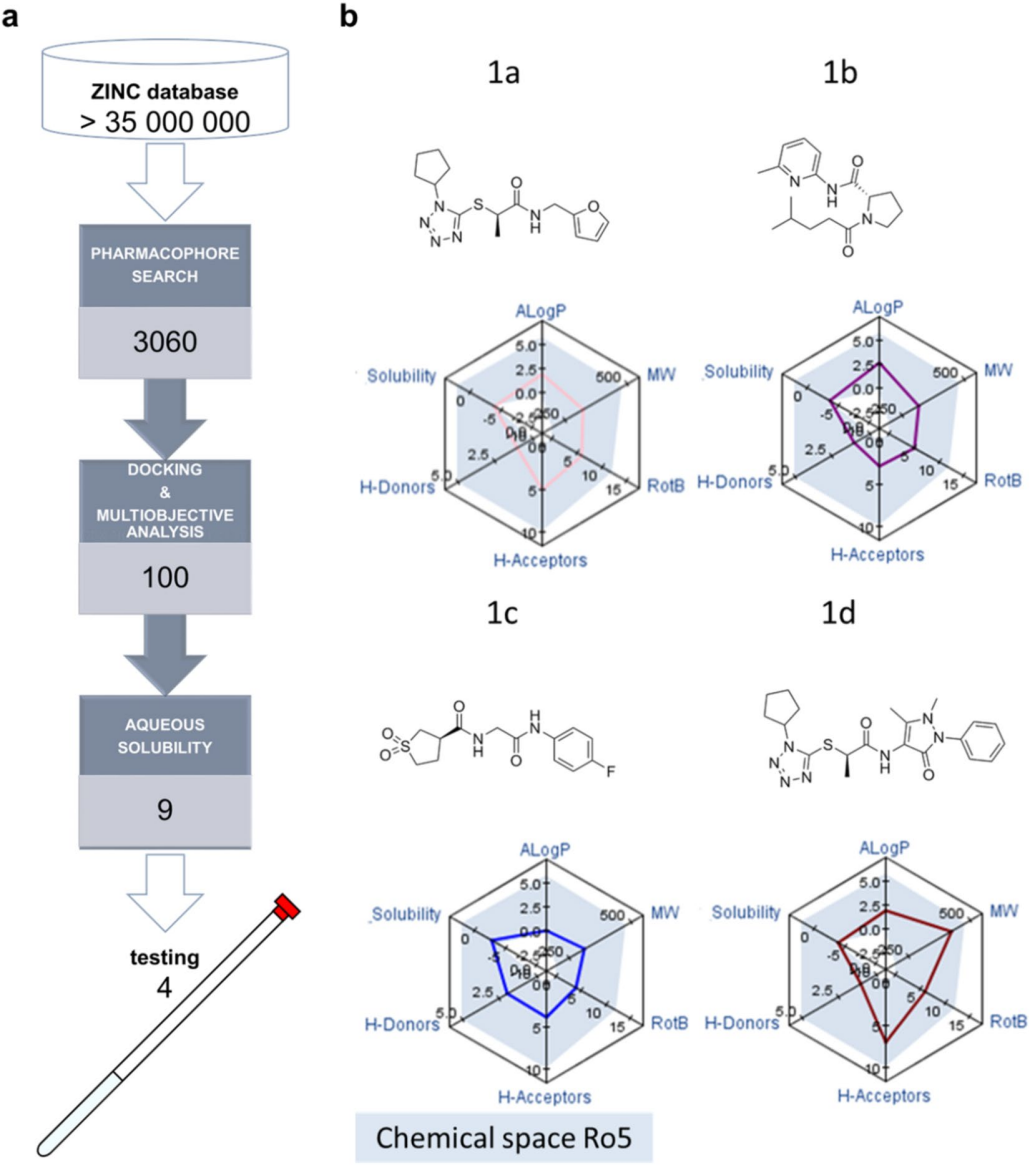
(**a**) General scheme for the virtual screening process, 3060 molecules were selected based on a pharmacophore model, docked and selected on the basis of multiobjective analysis using several parameters as ligand efficiency and drug-like molecular properties. (**b**) Selected hits for testing and respective molecular properties mapped in a radar plot. The shaded area in the radar plot corresponds to the chemical space of Lipinski’s rule of 5 for oral drugs.

The outcome of a virtual screening process is highly dependent on the library that is searched^12^. For this reason, we have chosen one of the largest *in silico* virtual libraries available, ZINC^13^. This library of over 35 million compounds was searched using ZINC Pharmer (Fig.  2a)^14^. The search was made using a pharmacophore model based on the SxIP motif for molecules that matched the favourable interactions between SxIP and EB1. This approach was selected to expedite the virtual screening process through the identification of virtual hits that fit the pharmacophore model, retaining a smaller set of compounds that will be subjected to more rigorous and time consuming virtual screening approaches such as docking (vide infra).

Eight pharmacophore points were defined using ZINC Pharmer (Fig. 1d). Due to the polar interactions observed for Ser5477 (described in the previous section), two hydrogen bond acceptors were defined for this residue. Three hydrophobic centroids were defined for Ile5479 and Pro5480 since these residues clearly make hydrophobic contacts. Lys5478 was not utilised for the definition of a pharmacophore model since it appears to make contacts outside the hydrophobic pocket (Fig. 1c). Additionally, the backbone amine for Ile5479 and carbonyl oxygen atoms for both Ile5479 and Pro5480 were included as hydrogen bond acceptor and donors, respectively. The use of all these pharmacophore features in one query did not yield any virtual screening hits from ZINC Pharmer and therefore a comprehensive search approach was used, searching for all 71 possible combinations of seven, six or five pharmacophore points. The total number of hit molecules from these combined searches was 40006 molecules. In order to select molecules which had the best overall geometric match to the pharmacophore query, we applied a Root Mean Square Deviation (RMSD) filter, removing molecules with RMSD larger than 0.5 Å, resulting in 3933 molecules. ZINC Pharmer provides different conformations of the same molecules, and duplicate molecules were removed, reducing the number to 3060 unique compounds. We then used GOLD^15–21^ to dock these 3060 compounds (Fig. 2a). We employed consensus scoring (GOLD has four scoring functions) to rank our compounds as previous work has shown that the false positive rate was reduced when compared to single scoring procedure^22^. In this approach, the best docked pose of each compound is re-evaluated with multiple scoring functions, with only the top scored compounds common to each scoring function identified as candidates for testing^23^.

The simultaneous prediction and optimisation of both binding affinity and molecular properties, such as aqueous solubility, is often challenging. Pareto-based methods are capable of optimising numerous properties simultaneously and can be used to make a balanced selection of compounds with the optimal overall profile^24^. Consequently, molecules, which were primarily docked and scored, were ranked based on a multi-objective selection using docking results and desirable molecular properties for a drug candidate – *i.e*. “Lipinski Rule of 5”^25^. This multi-objective selection ranks higher compounds that have optimal values in all the parameters considered, allowing for a balanced rank based on a variety of properties. From the 3060 compounds, the 100 best ranked molecules were selected based on their rank from the Pareto analysis (Fig. 2a), *i.e*. selecting compounds which are predicted to bind strongly to EB1 whilst having favourable physicochemical characteristics. These 100 top compounds were docked again (using higher search efficiency) and ten poses predicted for each molecule. All poses were visually inspected and key contacts predicted to occur between the ligand and the protein were analysed. These 100 molecules displayed hydrogen bonds with the following residues as was sought: Arg214, Arg222, Glu213, Gly252, Leu221, but more frequently with Ala248, Gln229 Glu225, Leu246, Lys220, Phe218, Pro256, Tyr217, Tyr247 and Val254. All these residues are located at the SxIP binding site of EB1 (Fig. 1c). RMSD values for the ten docking poses were calculated and molecules which had small RMSD values were prioritised due to the consistency of the prediction. At this stage we excluded compounds with predicted aqueous solubility values lower than 10^−4^ M (this value was chosen based on concentrations needed for the NMR measurements), yielding a total of nine molecules. In order to make our final selection a thorough visual analysis was performed of the docking poses and four molecules were selected for testing (Fig. 2a).

### NMR ligand screening

Ligand screening was performed for compounds **1a-1d** (Fig. 2b) using ^1^H, ^15^N heteronuclear single quantum coherence (HSQC) spectra of uniformly ^15^N-labelled EB1_191–260_ (EB1c) recorded in the absence and presence of the compounds. The ^1^H and ^15^N resonances were fully assigned using complementary pairs of triple resonance NMR spectra - CBCA(CO)NH/HNCACB for Cα and Cβ and HNCO/HN(CA)CO for CO connectivities.

**Figure 3:**
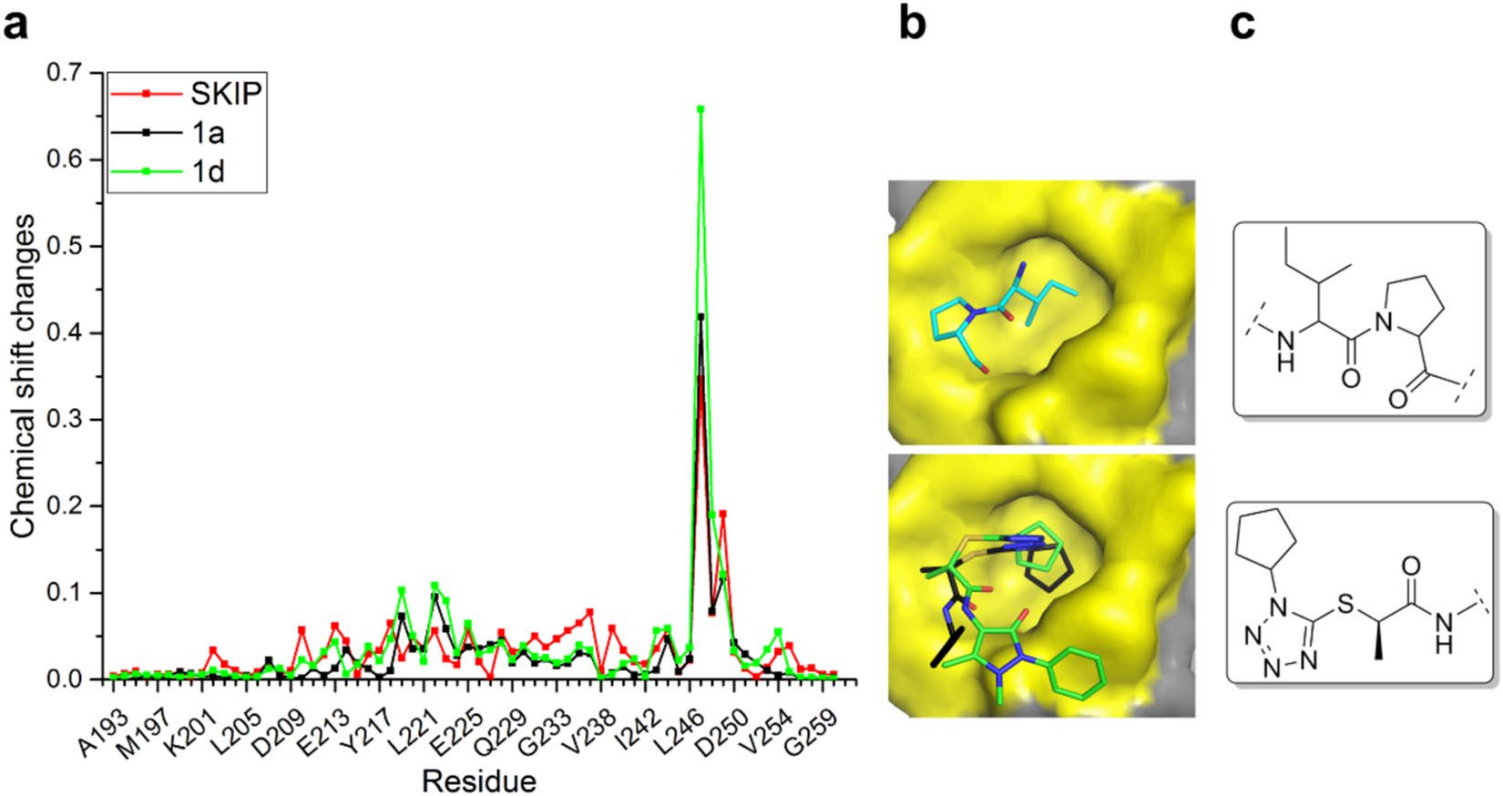
Comparison between the IP mimetic compounds and the natural SxIP motif. (**a**) Chemical shift changes plot for SKIP peptide (red), 1a (black) and 1d (green). (**b**) 3D model for the IP motif and the IP motif mimetic. The IP motif three dimensional representation is based on the crystal structure 3GJO. The IP mimetic compounds 1a (black) and 1d (green) are shown in the binding poses predicted by our docking studies using 3GJO structure as the EB1c model. In both representations the C-terminus tail was removed for clarity. (**c**) 2D structure of IP motif and IP mimetic scaffold.

Ligand-induced chemical shift perturbations (CSPs) in NH resonances on addition of the ligand were used as an indication of ligand binding and location of the binding site. All compounds were shown to induce CSPs in the backbone of EB1c, with **1a** and **1d** displaying the largest spectral changes (Supplementary Figs S2 to S6). No broadening was observed for the NH cross-peaks throughout the titration, indicating a fast exchange between the free and bound state and, therefore, weak interaction. Overall, the NH resonance for Tyr247 is the most affected upon ligand binding, with ∆δ = 0.42 ppm for 1a and ∆δ = 0.66 ppm for 1d at the final titration points). For compound **1a**, _247_YAT_249_ and _219_GKLR_222_ are the main regions where chemical shift changes are located. **1d** shows a very similar CSP pattern, with additional change in Val254 resonances (∆δ = ppm). The CSPs caused by compounds **1a** and **1d** were compared with the perturbations caused by the natural peptide sequence, SKIP (Fig. 3a). Similarly to the compounds 1a and 1d, largest shift changes were observed for Tyr247 (∆δ = 0.35 ppm), and the majority of CSPs were mapped to the _247_YAT_249_ region. Additional CSPs were observed for Glu213 (∆δ = 0.062 ppm), Arg222 (∆δ = 0.056 ppm), Phe218 (∆δ = 0.065 ppm), and Glu225 (∆δ = 0.056 ppm), located at the binding site (Fig. 1c). Despite a similar CSP profile, larger perturbations are observed for both **1a** and **1d** when compared with the SKIP peptide (Fig. 3a), suggesting stronger interaction for the small molecules.

NMR titration curves show significant deviation from a linear dependence at high ligand excess that allows for K_D_ estimation by fitting the curve into the two-state exchange model (Supplementary Fig. S8). In agreement with the CSP amplitudes, the estimated K_D_ value is highest for the SKIP peptide (14 ± 1.9 mM), followed by compound **1a** (10 ± 3 mM) and finally compound **1d** (6 ± 1 mM). Thus both molecules interact with the SxIP site with affinities that are slightly higher than the affinity of the natural SKIP fragment of the natural ligand. Both of the best binding compounds **1a** and **1d** share the same scaffold (Fig. 2b). Analysis of the docking poses and comparison with the IP motif of MACFp1 in the crystal structure of the complex (Fig. 3b) indicates that this scaffold may act as an IP motif mimetic, where the hydrophobic side chain of the isoleucine is replaced by a cyclopentyl ring, and the hydrophobic proline ring is replaced by a methyl group. The tetrazole moiety acts as spacer between both hydrophobic regions rigidifying the scaffold (Fig. 3c).

### Ligand binding affects binding site shape

To validate the docking predictions and to gain a structural insights into the binding mode of the IP mimetic scaffold we determined the three dimensional NMR structure of EB1c bound to compound **1a**. We also determined the structure of EB1c in the uncomplexed free state to identify structural changes upon ligand binding and to facilitate structure determination of the complex. The NMR spectra of EB1c showed good chemical shift dispersion, allowing complete resonance assignments using triple-resonance experiments. However, structure determination presented a significant challenge due to the leucine zipper arrangement of the protein dimer, with leucine and valine aliphatic side-chains making contacts both within the monomer and across the dimer interface (Fig. S9)^26^. This meant that residues close to the dimer interface are particularly difficult to assign since intermonomer peaks between equivalent residues are indistinguishable from intra-residue peaks and intermonomer peaks between equivalent protons of different monomers are on the diagonal and thus unmeasurable^27^. To identify intermolecular contacts for structure determination we used 3D filtered ^1^H-^13^C-NOESY-HSQC experiment recorded for a mixed ^13^C,^15^N/^12^C,^14^N EB1c dimer. The structure of the free protein was determined from 1652 intramolecular and 634 intermolecular distance restraints, supplemented by 64 dihedral angle restrains derived from chemical ^13^C-chemical shift values. In the presence of compound **1a** we detected 75 intermolecular NOEs in the 3D filtered ^1^H-^13^C-NOESY-HSQC that were additionally used for calculating structure of the complex. Statistics of the structure determination are presented in Supplementary Table S1.

**Figure 4:**
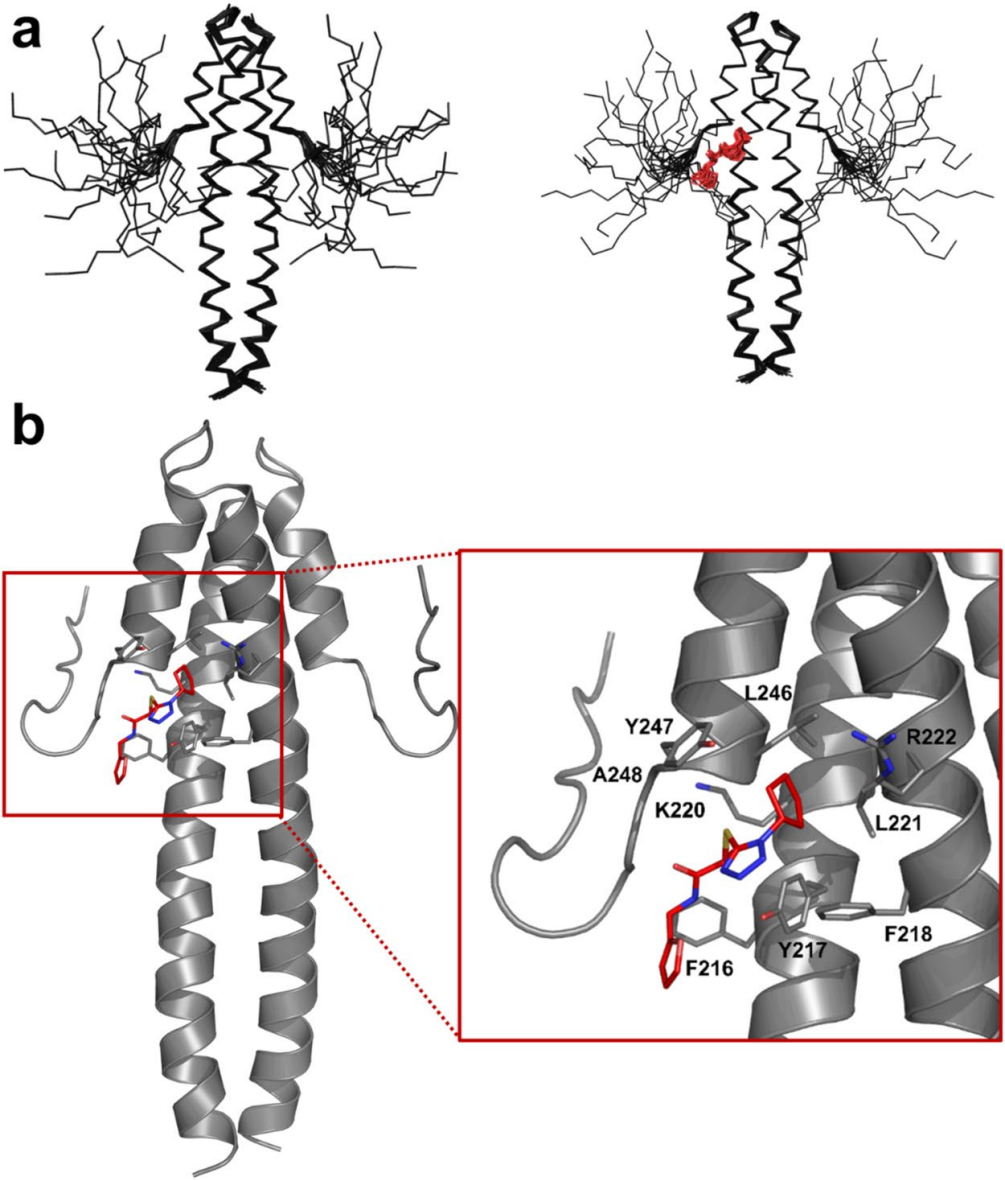
(**a**) Overall representation of an ensemble of 20 structures of EB1c domain (residues 191–260) in the free form (left hand side) and bound to 1a (right hand side). (**b**) Lowest energy structure of the complex with molecule **1a**.

The long N-terminal helix (residues 191–230) forms a classical leucine zipper in the region 191–214 and then part of the 4-helix bundle of the EBH domain in association with the C-terminal helix (residues 237–248). The packing of the helices is stabilised by the extensive network of hydrophobic interactions characteristic for coiled-coils (Supplementary Fig. S9)^26^. The C-terminal region of EB1c (residues 248–260) is unstructured and dynamic – Fig. 4a. Only intra-residue and sequential NOE contacts were observed in this region (Supplementary Fig. S10) and the intensities of the backbone ^1^H,^15^N-HSQC cross-peaks dramatically increased for the corresponding residues, reflecting the increase in dynamics.

**Figure 5:**
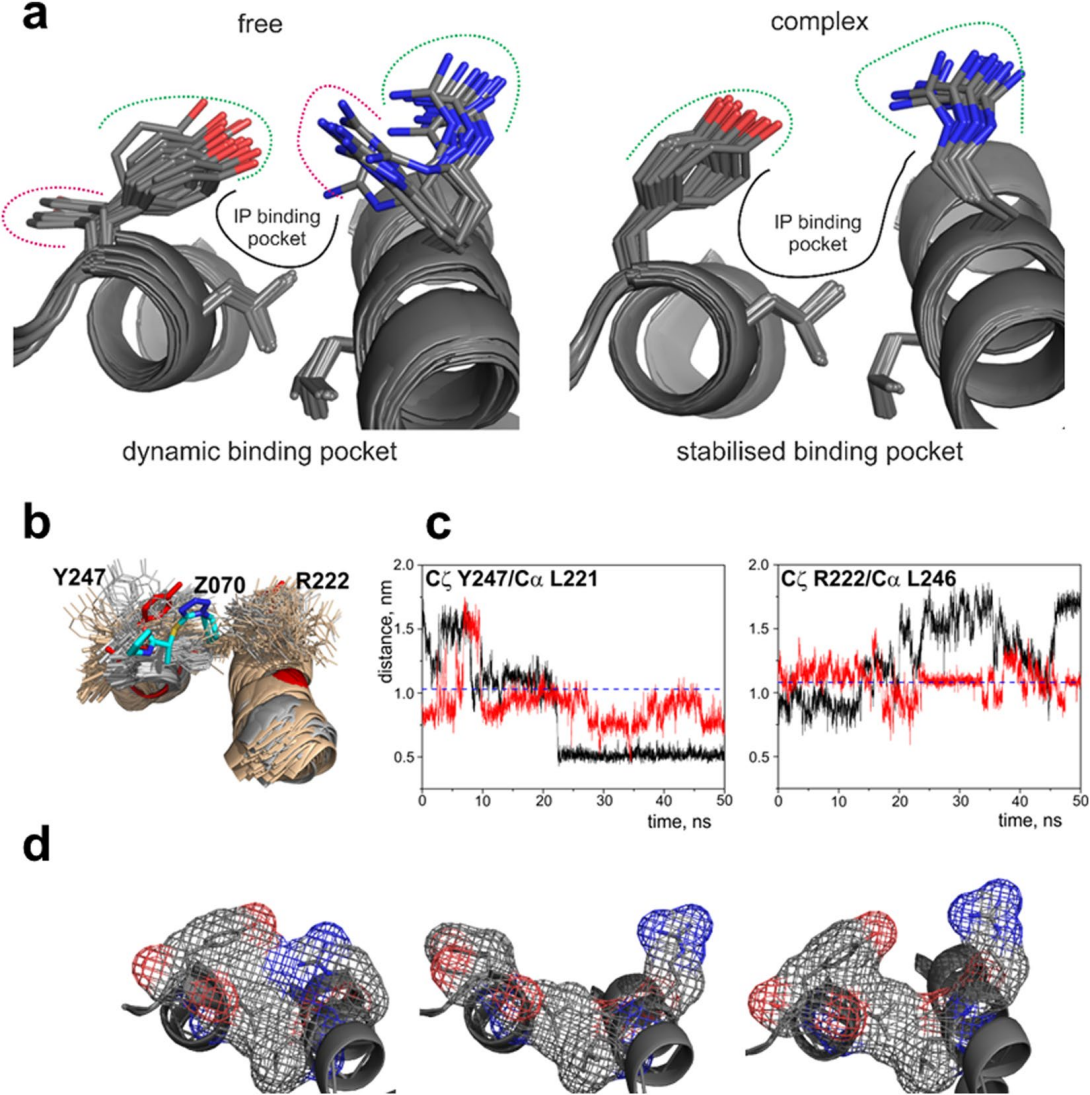
(**a**) View of the IP binding site of EB1 for the ensemble of 20 NMR structures in the free form – left hand side, and the ensemble of 20 NMR structures bound to **1a** – right hand side. (**b**) Superposition of 100 structures extracted from a 50 ns MD simulation starting from the open (light orange) and closed (grey) orientations of Tyr247 side-chain, and the lowest energy NMR structure of EB1c (red) in complex with **1a** (cyan). Structures were superimposed on the Cα atoms of residues in the helical regions Phe218-Glu232 and Leu239-Tyr247 of the model of the complex. (**c**) Variations of the distances between Cζ of Tyr247 and Cα of Leu221 (left), and Cζ of Tyr247 and Cα of Leu246 (left) over the 50 ns MD simulations starting from the structure of the open (black) and closed (red) orientations of Tyr247. The corresponding distances measured in the lowest energy NMR structure of EB1c are represented by the dashed lines. (**d**) Effect of different conformations observed for Arg222 and Tyr247 in solution for free EBH domain on the shape and size of the binding pocket.

The absence of the ligand has a strong effect on the orientation of the solvent-exposed side-chains of Arg222 and Tyr247 at the opposite sides of the IP binding pocket. In the free protein both side-chains have variable orientations across the ensemble of the calculated structures (Fig. 5a, Supplementary Fig. S11a). We observed NOE contacts between the aromatic ring of Tyr247 and Val243, Asp244, Ile245, Leu246, and Ala248 in the immediate proximity of Tyr247, compatible with all the detected orientations of the side-chain. However, we did not detect NOEs between Tyr247 and Gln240 or Glu225, expected for two of the lowest energy structures of the protein (Supplementary Fig. S12). In addition, the cross-peak intensities of the side-chain signals of Tyr247 in ^1^H,^13^C-HSQC spectrum were much higher than the signals intensities of Tyr217 that is immobilised inside the hydrophobic core of the EB1 dimer and has a fixed orientation, indicating mobility of the Tyr247 side-chain. Similarly, orientation of Arg222 side-chain varied across the calculated structures, in agreement with the absence of non-sequential NOEs contacts. The cross-peak intensities of the side-chain signals of Arg222 were much higher than of the corresponding signals of Arg214 and Arg241 that have preferred orientations due to the proximity of negatively charged groups of Glu211 and Glu225, respectively, across the helical turns, supporting mobility of Arg222 side-chain.

To estimate the degree of conformational freedom for Arg222 and Tyr247 side-chains we conducted molecular dynamics (MD) simulations in the absence of NOE restrains, starting from the two lowest-energy NMR structures with different orientations of Tyr247 aromatic ring (Fig. S12). The MD trajectories showed large variation in the orientations of Arg222 and Tyr247 side-chains, as expected of the solvent-exposed side-chains (Fig. 5b), with the residue-specific root mean square fluctuations (RMSF) for these residues high and similar to other solvent-exposed side-chains of the helical regions (Supplementary Fig. S11b). We used distances between Cζ atoms of Arg222 and Tyr247 side-chains and CA atoms of L246 and Leu221 across the binding site (respectively) as measures of the side-chain locations relative to the binding site. For both residues, distances showed large variations (Fig. 5c), corresponding to fluctuations between side-chains pointing into the binding site and out, into solution. Thus the MD simulations support the NMR-based conclusion that in the free protein the side-chains of Arg222 and Tyr247 are dynamic, due to the lack of any specific interactions that would stabilise their orientation.

In contrast to the solution structures, crystal structures of free EB1c (PDB codes 1YIG^28^ and 1WU9^29^) show fixed orientation of the Arg222 and Tyr247 side-chains in conformations that are similar to some of the conformations observed in the NMR ensemble (Supplementary Fig. S13). However, the NMR data and MD simulations do not support this preferential orientation, suggesting that the fixed orientations of the side-chains are induced by crystallisation.

The structure of the EB1c/**1a** complex is consistent with the docking prediction. The cyclopentyl ring is inserted into the IP binding pocket in the orientation similar to the orientation of the Ile side-chain (Fig. 4b), supported by extensive NOE contacts to Tyr217, Phe218, Leu221, Arg222, Leu246 and Tyr247 at the hydrophobic binding site (Supplementary Fig. S14). The methyl group of the compound has NOE cross peaks to Tyr217, Phe218 and Tyr247 of the IP binding pocket, as well as to Phe216 and Thr249 in the coiled-coil region. No intermolecular NOEs were observed for the oxazole moiety, suggesting that it is not involved in the interaction with the protein. The C-terminus of EB1c remains dynamic (Fig. 4b), as the only intermolecular NOE contact was detected for Thr249 located near the end of the helical region. The binding pocket in the complex of EB1c with 1a is maintained throughout the whole ensemble of calculated structures and has similar configuration to the binding pocket of the peptide ligand (Fig. 5a, right panel). The orientations of side-chains of Arg222 and Tyr247 are well defined, forming the walls of the binding pocket and making contact with the ligand. This suggests that the ligand binding stabilises the configuration of the binding pocket, restricting the mobility of the side-chains.

The majority of the NMR structures of free EB1c had either open or at least partially closed configuration of the binding pocket (Fig. 5d), compared to the structure of the complex. MD trajectories revealed that the orientations of Arg222 and Tyr247 side-chains corresponding to their position in the complex are adopted only in transition between open and closed orientation (Fig. 5b). For Tyr247 the closed form persisted longer than the open form due to the contacts with surfaces of the helices. At the same time, the average energy of the protein remained unchanged on the transition between the close and open forms, suggesting that the persistence of the closed form was caused by the steric restrictions of the Tyr247 ring motion rather than formation of favourable contacts. In the closed form the orientation of the Arg222 and Tyr247 side-chains would prevent ligand from entering the binding site; however the binding site opening occurs spontaneously. We thus conclude that the interaction follows classical conformation selection mechanism, with the affinity of the interaction reduced due to the dynamics of the binding site in the free form.

Exploration of the potential effects of the binding pocket dynamics were achieved by re-evaluating small molecule binding to EB1c by ligand cross-docking^30^ to the variety of experimental EB1c structures with different binding site conformations. This approach is essential when targeting dynamic regions and increases the chances to find near-native solutions^31^. We thus re-docked the four experimentally tested molecules using the ensemble of 20 NMR structures for the free form, as well as the crystal structures of free EB1 (1YIG and 1WU9) and EB1c bound to a SxIP containing peptide (3GJO).

### Reassessment of the docking results based on structural data

The average docking score for the NMR ensemble for compound **1d** was highest [64], followed by **1a** [53], **1c** [48] and **1b** [46]. This ranking is in accordance with the *in vitro* screening, where **1d** is the best binding compound, followed by **1a**; compounds **1c** and **1b** have much weaker interactions. In contrast, when docked to the crystal structure of complex with the peptide (3GJO), compound 1b had a docking score of 59 that was higher than for compound **1a** [55], in clear disagreement with the experimental measurements (Supplementary Fig. S15). The modelling indicates that compounds **1a** and **1d** can interact with a wide range of states, where the binding site is partially formed, while **1b** and **1c** only bind to the fully formed binding pocket. In agreement with this, while all compounds had low scores when docked to the open binding pocket of the crystal structures of the free EB1c (1YIG and 1WU9), the scores of the compounds **1a** and **1d** were significantly higher than the scores of the other two compounds (Supplementary Fig. S15). The docking results provide an explanation of why compound **1b**, that apparently fits the binding pocket well, shows negligible interaction with EB1c. This compound can only interact with an extremely small population of EB1c where the binding site is fully formed spontaneously, while compounds **1a** and **1d** interact reasonably well with the majority of the configurations of the binding pockets, potentially inducing further binding pocket changes after an initial docking. This modelling analysis, performed after the measurements were made, show closer agreement with the experimental results and suggest that the use of solution NMR structures and cross-docking can be a powerful tool in drug design for dynamic regions.

## Discussion

Modulating the interaction between EB1 and SxIP proteins using small molecules can have huge potential as this interaction allows for the localisation of SxIP proteins to the microtubule plus ends and some of these proteins have been shown to be directly related to diverse cancer diseases. We have identified a molecular scaffold that mimics the SxIP motif essential for the recognition of the SxIP proteins by the EBH domain of EB1. We have also demonstrated that the scaffold binds to the SxIP binding pocket with similar affinity and spatial orientation to that of the natural ligand.

For scaffold identification we used a targeted approach based on combination of pharmacophore-driven compound selection followed by a docking analysis and multiparameter compound prioritisation that can reduce expensive and time-consuming experimental screening of large numbers of compounds, potentially even replacing screening at the first stage of lead compound selection. The interaction of the selected molecules was shown experimentally by NMR spectroscopy and this provided information on molecular interaction critical for ligand recognition. Structure elucidation of EB1c in the free and bound forms allowed us to identify two residue side-chains, Arg222 and Tyr247, at the binding site with highly dynamic behaviour in the free protein form that had a strong effect on the geometry of the binding pocket.

Analysis of previously published X-ray crystal structure obtained for the bound form of EB1c (PDB code 3GJO^5^) indicates that Arg222 and Tyr247, adopt different positions from the ones observed for the free form (Supplementary Fig. S13). The change in the orientation of the Arg222 and Tyr247 side-chains clearly affects the shape of the binding IP pocket (Fig. 5d and Supplementary Fig. S13b). In the absence of a ligand the binding pocket is dynamic, changing from the fully open state to the fully closed state (Fig. 5d) with the open state characterised by Tyr247 side-chain pointing outwards and Arg222 side-chain flattened against the surface of the helix leading to the absence of one of the outer walls of the binding pocket. This structure is stabilised in the crystallised free EB1c (Supplementary Fig. S13b). In the closed form, side-chains of Arg222 and Tyr247 point towards each other, completely blocking the binding site. This closed form is present in the NMR ensemble (Fig. 5d, left panel). When both side chains point away from each other the binding site is open but does not have the adequate shape to accommodate the IP motif/ IP mimetic – Fig. 5d, middle panel, being too flat. The binding pocket is fully formed in the crystal structure of the complex (Supplementary Fig. S13b, right panel) and some of the structures of the NMR ensemble have similar orientations of the side-chains (Fig. 5d, right panel).

To explore further how the dynamics of the binding pocket affect ligand binding we used the predicted affinity obtained through the use of cross-docking methods and showed that it coincides with the experimental data obtained. This is a demonstration that docking to the NMR ensemble provided a much more reliable prediction of the binding propensity than the docking to the fully formed binding site of the complex, *i.e*. crystal structure. This method allowed us to address the conformational dynamics of the binding pocket in ligand identification, overcoming the limitations of docking in terms of side chain dynamics, without the time and resource consuming molecular dynamic simulations.

The understanding we now have on the EB1 binding pocket was only possible through the use of solution NMR and we report the first structure of EB1 complexed with a small molecule SxIP mimetic. This information, in addition to the computational methodology developed within this project, can now be used to identify higher affinity ligands. We believe that our integrated computational and NMR approach is generally applicable in the design of inhibitors targeting other dynamic protein-protein interaction sites. The improved differentiation between compounds with docking to the NMR ensemble highlights benefits of using solution structures in docking approaches.

## Methods

### Virtual Screening methods

The crystal structure of a complex formed between the C-terminal of EB1 lacking the last eight C-terminal residues (EB1c∆8) and a 30 residue peptide derived from the C-terminal of human MACF2 (MACFp1)^5^, with code 3GJO, was downloaded from the RSCB Protein Data Bank (PDB)^32^.

#### Pharmacophore search

EB1 crystal structure (3GJO) was loaded into ZincPharmer (zincpharmer.csb.pitt.edu)^14^, using Load Features option. Selected options included definition of Max RMSD value, molecular weight and number of rotatable bonds.

#### Docking Protocol

The asymmetric unit of the crystal structure of the EB1 complex with the MACFp1 peptide (3GJO) is composed of two homodimers, each one with two binding sites and two ligands. For the molecular docking studies just one of the homodimers was used. Protein-ligand molecular docking at the EB1 binding site was performed using GOLD 5.0.1^15–21^. EB1 crystal structure (3GJO) was loaded (pdb format) into GOLD using the wizard menu. Hydrogen atoms were added to the protein using the protonation rules file provided with GOLD When specified HOH19 water molecule was extracted for inclusion in docking calculations. All other crystallographic water molecules were removed. Hydrophobic and hydrogen bonds constraints were used or not depending on the docking protocol. MACFp1 ligand was later loaded and used to define the binding site, together with all atoms around the ligand within 6 Å. Ligand file(s) containing the compound(s) to be docked were loaded in sdf format. As standard, for each ligand 10 GA runs are performed. Scoring function *i.e*. GOLDScore, was selected. For rescoring purposes then select an additional scoring method *e.g*. ChemScore. Option for early termination turned off and the search efficiency set to 200%.

#### Screening and selection of target molecules

The following components of molecular properties were calculated using Pipeline Pilot Professional Client 8.5^33^: ALogP, logD, Solubility, Surface Area and Volume, Molecular weight, Num H Acceptor Donors and Molecular Property Counts. Ligand efficiency was calculated using KNIME 2.6.3^34^ with Math Formula node with the formula “score”/number of atoms for each scoring function. The results were ranked in an ascendant order and a new column with a ligand efficiency ranking was added to the SD file. Knime 2.6.3 was used for multi-objective analysis, using the node Pareto Ranking to rank the different parameters required for the analysis. “Align molecules” from Pipeline Pilot were used for RMSD calculation between different docking poses.

### Synthesis

All the compounds were purchased from Enamine or MolPort, apart from compound 1b, which was synthesised *in-house*.

**Compound 1b** Proline benzyl ester (556 mg, 2.3 mmol), 4-methyl valeric acid (327 µL, 2.6 mmol, 1.1 eq.) and HOBt (398 mg, 2.6 mmol, 1.1 eq.) were dissolved in 30 mL of anhydrous DCM. After 10 min EDCI (500 mg, 2.6 mmol, 1.1 eq.) were added, followed 10 min later by DIEA (881 µL, 5.06 mmol, 2.20 eq.). The reaction was allowed to stir overnight. The DCM was removed and the residue was dissolved in EtOAc. The organic layer was washed three times with 1 N HCl, three times with saturated NaHCO_3_ and three times with brine. The organic layer was dried with Na_2_SO_4_, filtered through filter paper, and concentrated. The product was purified by column chromatography. Fractions were analysed by TLC, concentrated and characterized. 411 mg of product were obtained as colorless oil (59% yield).

^1^H NMR (400 MHz, CD_3_OD)δ: 7.35 (br d, 5H), 5.14 (d, 2H, *J* = 5.7 Hz), 4.47 (m, 1H), 3.63 (m, 2H) 2.36 (m, 2H), 1.96 (m, 4H), 1.58 (m, 1H), 1.47 (m, 2H) 0.91 (dd, 6H, *J* = 6.6, 0.98 Hz) *m/z* (LCMS, CI): found 304.19 (M+H)^+^, C_18_H_25_NO_3_, requires 303.18.

The ester was dissolved in MeOH in a Parr bottle. The bottle was flushed with argon and 0.050 eq. of 10% Pd/C. The Parr bottle was placed on a Parr hydrogenation apparatus and subjected to three charge/purge cycles with H_2_. The reaction was then charged with 5–10 bar hydrogen and shaken. After 4,5 hours no starting material was observed. The product was dried and 266 mg were obtained as white crystals (92% yield).

^1^H NMR (400 MHz, CD_3_OD)δ: 4.42 (m, 1H), 3.62 (m, 2H), 2.36 (m, 2H), 2.24 (m, 2H), 2.02 (m, 2H), 1.61 (m, 2H), 1.52 (m, 2H), 0.93 (d, 6H, *J* = 6.52 Hz) CHN analysis: C 59.90%, H 8.58% and N 6.62%. C_11_H_19_NO_3_ *m/z* (LCMS, CI): found 214.14 (M+H)^+^, requires 213.14.

266 mg of the compound obtained in the previous step and 6-methyl-2-aminopyridine (118 mg, 1.25 mmol, 1 eq.) were dissolved in 7 mL of DCM. The solution was cooled to 0 °C, and then DCC (516 mg, 2.5 mmol, 2 eq.) was added. The solution was stirred at 0 °C for 2 h and at room temperature for another 16 h. Then the solution was placed in the refrigerator (4 °C) for 2 h, and the white solid was filtered. After removal of solvent under reduced pressure, the residue was purified by column chromatography. 102 mg of a white solid were obtained (29% yield).

^1^H NMR (400 MHz, CDCl_3_)δ: 9.68 (br s, 1H), 8.29 (d, 1H, 4.0 Hz), 8.15 (d, 1H, 8.3 Hz), 7.66 (td, 1H, *J* = 7.7, J = 1.80 Hz), 7.00 (dt, 1H, *J* = 5.3, *J* = 1.80 Hz), 4.79 (dd, 1H, *J* = 8.19, *J* = 1.86 Hz), 3.63 (m, 1H), 3.50 (m, 1H), 2.50 (m, 1H), 2.36 (t, 2H, *J* = 7.5 Hz) 2.06 (m, 4H), 1.60 (m, 2H), 0.91 (d, 6H, *J* = 5.92 Hz) ^13^C NMR (100 MHz, CDCl_3_)δ: 175.70, 171.31, 149.27, 139.58, 120.97, 115.45, 62.19, 49.00, 34.80, 34.07, 29.26, 28.66, 26.47, 23.81, 23.73 CHN analysis C 66.02%, H 8.01%, N 13.62%, C_16_H_23_N_3_O_2_ *m/z* (LCMS, CI): found 290.18 (M+H)^+^, requires 289.18 Total yield-16%.

### Protein expression and purification

Human EB1 (Uniprot code Q15691), residues 191–260, was cloned into pOPINS vector. The vector was introduced into BL21 Star^TM^ (DE3) competent cells using heat-shock transformation protocol. Isotope-labelled EB1 (^15^N, ^13^C) was expressed in 2M9 minimal medium. Purification was achieved by nickel affinity chromatography, followed by removal of the Sumo fusion protein and 6xHis tag using sumo protease. After new nickel purification to remove the 6xHis tagged sumo an extra ion exchange chromatography step was added to assure high purity.

### NMR experiments

NMR spectra were collected on Bruker Avance III 600 and 800 MHz spectrometers equipped with CryoProbes. Spectra were processed with TopSpin (Bruker) and analysed using CCPNmr Analysis^35^. ^1^H-^15^N HSQC ligand screening ^1^H-^15^N- Heteronuclear Single Quantum Coherence (HSQC) experiments for ligand binding screening were performed using 0.05 mM ^15^N-labelled EB1 in 20 mM phosphate pH 6.5, 50 mM NaCl, 0.5 mM TCEP, 0.02% (w/v) NaN_3_ in the absence or presence of ligands. To eliminate any small shifts induced by the small amounts of DMSO used to dissolve the compounds, DMSO concentration was kept constant in all experiments.

#### Resonance assignments

The backbone resonances were assigned using triple resonance experiments (HNCO, HN(CA)CO, HNCA, HNCACB and CBCACONH) measured for ^13^C-^15^N labelled EB1 using standard assignment protocols in CCPN Analysis.

#### Solution NMR structure determination

Side chain resonance assignments were obtained using HBHA(CO)NH, H(C)CH-TOCSY and (H)CCH-TOCSY experiments. Aromatic side-chains were assigned using 2D-NOESY and ^1^H-^13^C-resolved-NOESY-HSQC. The resonances of the ligands were assigned using ^13^C,^15^N-filtered 2D TOCSY and NOESY experiments. The structures were calculated using ARIA 2.2^27^ integrated with CCPNmr Analysis. Cross-peaks in the NOESY spectra were assigned automatically by matching the chemical shift values. Overall all spectra showed good resonance dispersion allowing for reliable assignments. However, the methyl region of the spectra is highly populated, creating significant signal overlap and assignment ambiguity caused by the presence of heptad repeats in the form *abcdefg*, where *a* is leucine and *e* valine, in EB1 sequence arrange into leucine zipper of the EB1 dimer. Therefore careful manual curation was used to resolve some of the ambiguous assignments of the methyl resonances. Cross-peaks intensities were converted into distance restraint using spin-diffusion correction protocol of ARIA 2.2. Inter- monomer restraints were identified from inter molecular NOEs detected in a ^13^C,^15^N-filtered/^13^C -resolved-NOESY-HSQC experiment measure for a mixed EB1 dimer made of ^13^C,^15^N-labelled and unlabelled monomers. The dimer was obtained by incubating equimolar solutions of unlabelled and labelled EB1c at 37 °C for 16 hours^11^. Dihedral angle restraints were generated from backbone ^13^C chemical shift values using DANGLE^36^ approach in CCPNmr Analysis. Hydrogen bond restraints were introduced for the helical regions of the protein identified from ^13^C chemical shift values and NOE contacts. Only minor ^1^H, ^13^C and ^15^N chemical shift changes restricted to the binding site were detected to the EB1c/1a complex, demonstrating that the conformation of the protein is not affected by the ligand binding. This was also supported by similar patterns of NOE cross-peaks. For this reason the final set of restraints from the structure calculation of the free EB1c, supplemented by the restraints obtained between the protein and the ligand were used to calculate the structure of the complex. The ligand-protein restraints were obtained from 3D and 2D ^15^N-^13^C-filtered NOESY experiments, and all EB1 free restraints incompatible with the intermolecular restraints for the complex were removed through an iterative process. All NOESY experiments had a mixing time of 200 ms. Structures were calculated using standard torsion angle dynamics (TAD) protocol implemented in ARIA 2.2. Symmetry between the protein molecules in the dimer was maintained using symmetry restraints implemented in ARIA 2.2 for dimeric structures. Single ligand molecule was used in the structure calculation of the EB1c/1a complex to allow automatic iterative resolution of ambiguity of restraints between ligand and proteins caused by the dimeric structure. This was not possible when two ligand molecules were used in the calculation, as the binding site incorporated residues from different monomers, leading to distorted high energy structures. The full complex was reconstructed by applying symmetry transformation to the ligand. To improve convergence of the structure calculation number of steps was increased and time-step decreased compared to the default parameters. Structures of the free protein were used as initial for the structure calculation of the complex with reduced maximum temperature of the simulated annealing stage.

The datasets generated during and/or analysed during the current study are available from the corresponding author on reasonable request.

### MD Simulations

MD simulations were performed in GROMACS 2016.4^37^ with AMBER99SB force field^38^ and the TIP3P water model^39^ following standard protocols. The NMR models were positioned into a cubic box of water with at least 1 nm from any edge. To compensate for charge of −11 per monomer at pH 7, 22 sodium atoms were added to the system. The system was energy minimised and then equilibrated at constant pressure of 1 bar to 1 ns. The temperature was maintained at 298 K throughout the simulations, corresponding to the temperature used in the NMR analysis. Trajectories were calculated from the lowest energy conformation of the NMR ensemble corresponding to the open and close position of Tyr247 side-chain. Trajectories were analysed using programs of GROMACS suite.

### Data Availability

The coordinates for the structure of free EB1 and EB1/1a complex have been deposited in the PDB, accession codes 6EVI and 6EVQ, respectively. The chemical shifts of free EB1 and EB1/1a complex have been deposited in the BMRB, accession codes 34191 and 34192, respectively.

## Electronic supplementary material

Supplementary information
