# Supplementary material for: Targeting SxIP-EB1 interaction: An integrated approach to the discovery of small molecule modulators of dynamic binding sites
Source: Sci Rep. 2017 Nov 14;7:15533. doi: 10.1038/s41598-017-15502-6 (PMC5686072; doi:10.1038/s41598-017-15502-6)
Supplement: Supplementary file 1 — Supplementary information [file 41598_2017_15502_MOESM1_ESM.pdf]

Supplementary information

Targeting SxIP-EB1 interaction: An integrated approach to the discovery of small molecule modulators of dynamic binding sites

T. B. Almeida,[1,2] A. J. Carnell,[1] I. Barsukov*,[2] N. G. Berry*[1]

[1] Robert Robinson Laboratories, Department of Chemistry, University of Liverpool, Liverpool L69 7ZD

[2] Institute of Integrative Biology, Biosciences Building, University of Liverpool, Crown Street, Liverpool L69 7ZB

* - Joint corresponding authors

Supplementary figures – S1 – S16
Supplementary table – S1

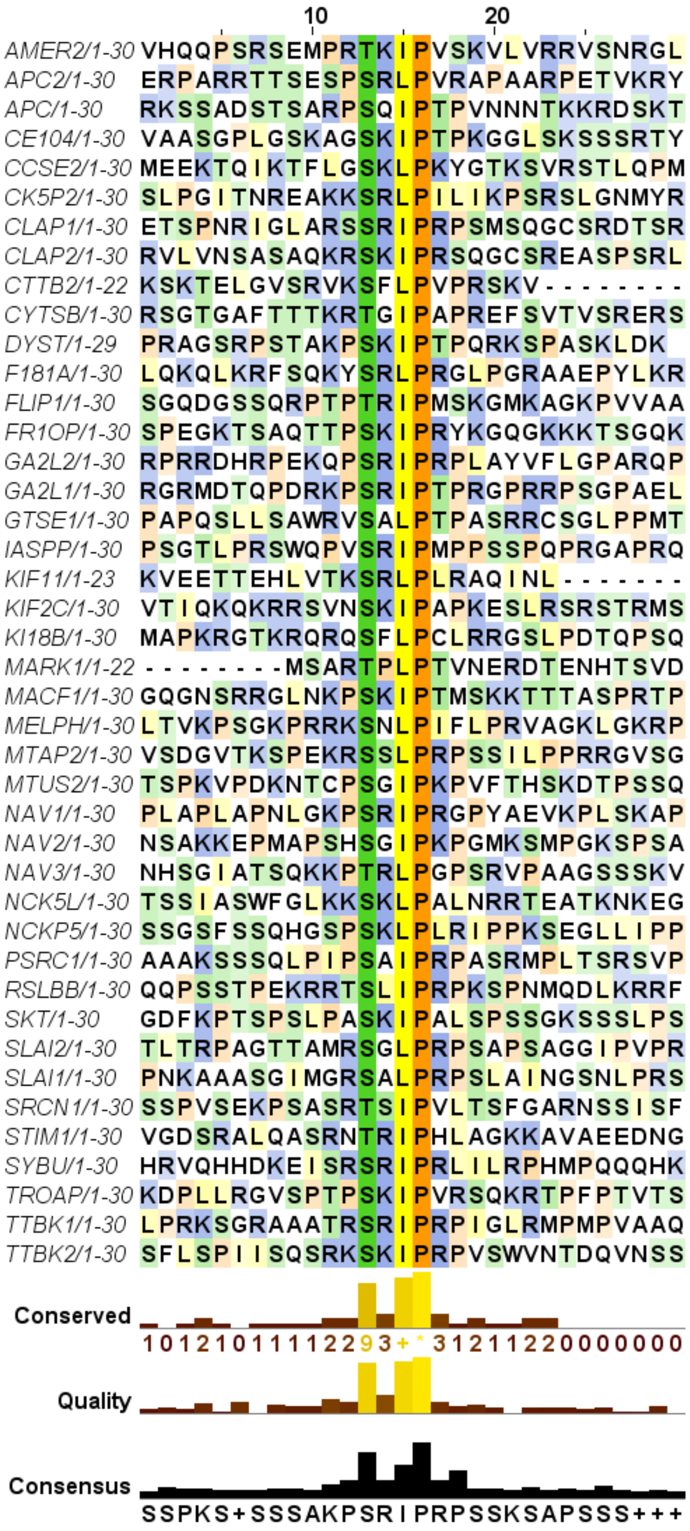

Figure S1 - Sequence alignment for known SxIP proteins based on a 30 residue sequence encompassing the identified SxIP motif. The sequence is colour coded as: positive charged residues (arginine and lysine) coloured in blue, serine and threonine coloured in green, hydrophobic residues (isoleucine and leucine) coloured in yellow and proline coloured in orange. Intensity of the colour indicates the higher conservation in that position. Figure was made using JalView 2.8.2.

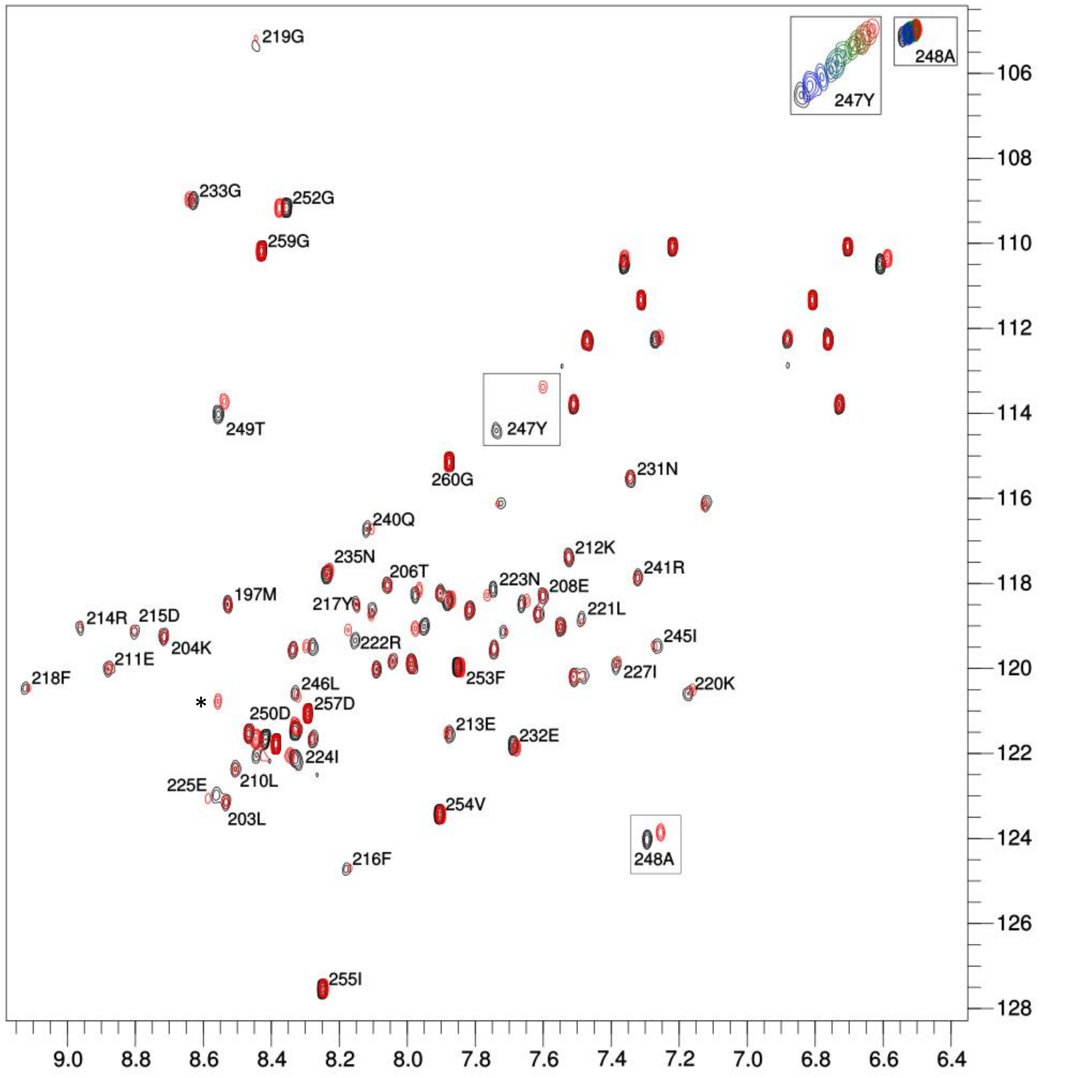

Figure S2 - Overlay of 2D ^{1}H-^{15}N – HSQC spectra recorded at 800 MHz – in black is presented EB1cΔ8 in the free form (50 µM) and in red the complex EB1cΔ8 (50 µM) – **1a** (5000 µM). The insets show superposition of signals of the most affected residues Tyr247 and Ala248 (highlighted by boxes) at the **1a** concentrations of 500, 1000, 1500, 2000, 2500, 3000, 3500, 4000, 4500 and 5000 µM used in the titration. Cross-peak marked by “*” corresponds to the ligand HN group at natural abundance, the is detectable only at high ligand concentrations

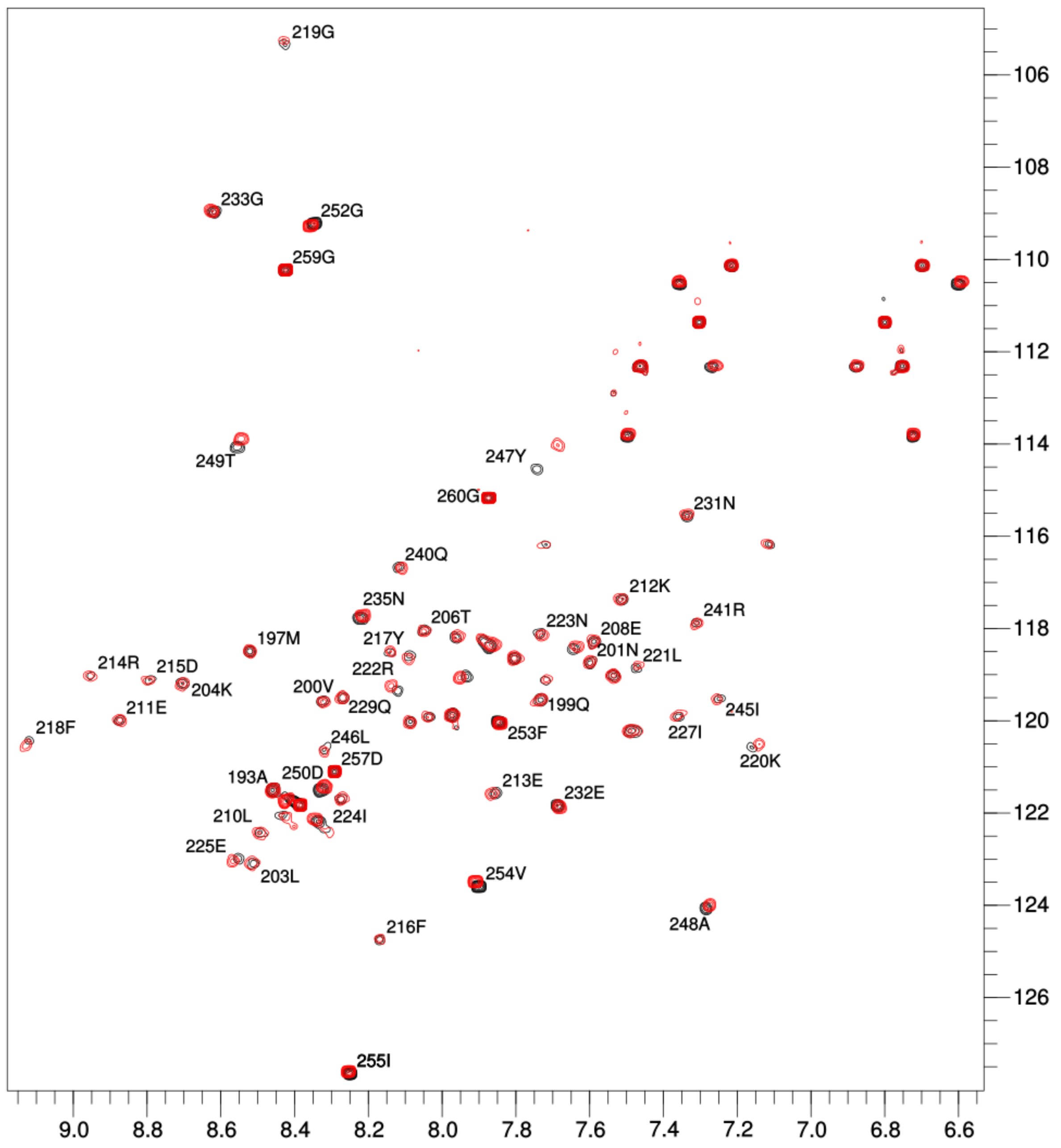

Figure S3 - Overlay of 2D ^{1}H-^{15}N – HSQC spectra recorded at 600 MHz – in black is presented EB1cΔ8 in the free form (50 μM) and in red the complex EB1cΔ8 (50 μM) – **1b** (5000 μM).

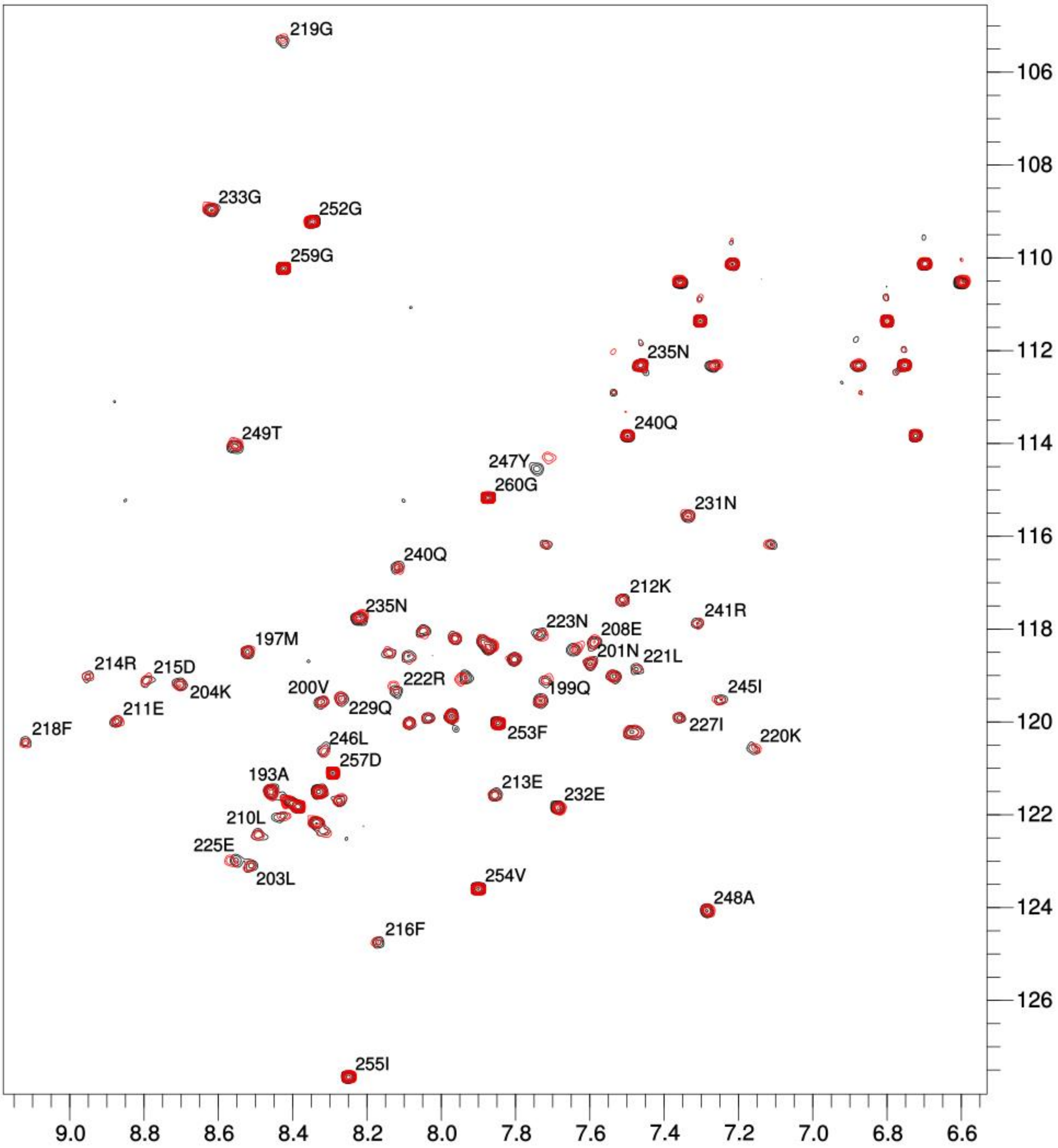

Figure S4 - Overlay of 2D ^{1}H-^{15}N – HSQC spectra recorded at 600 MHz – in black is presented EB1cΔ8 in the free form (50 µM) and in red the complex EB1cΔ8 (50 µM) – **1c** (5000 µM).

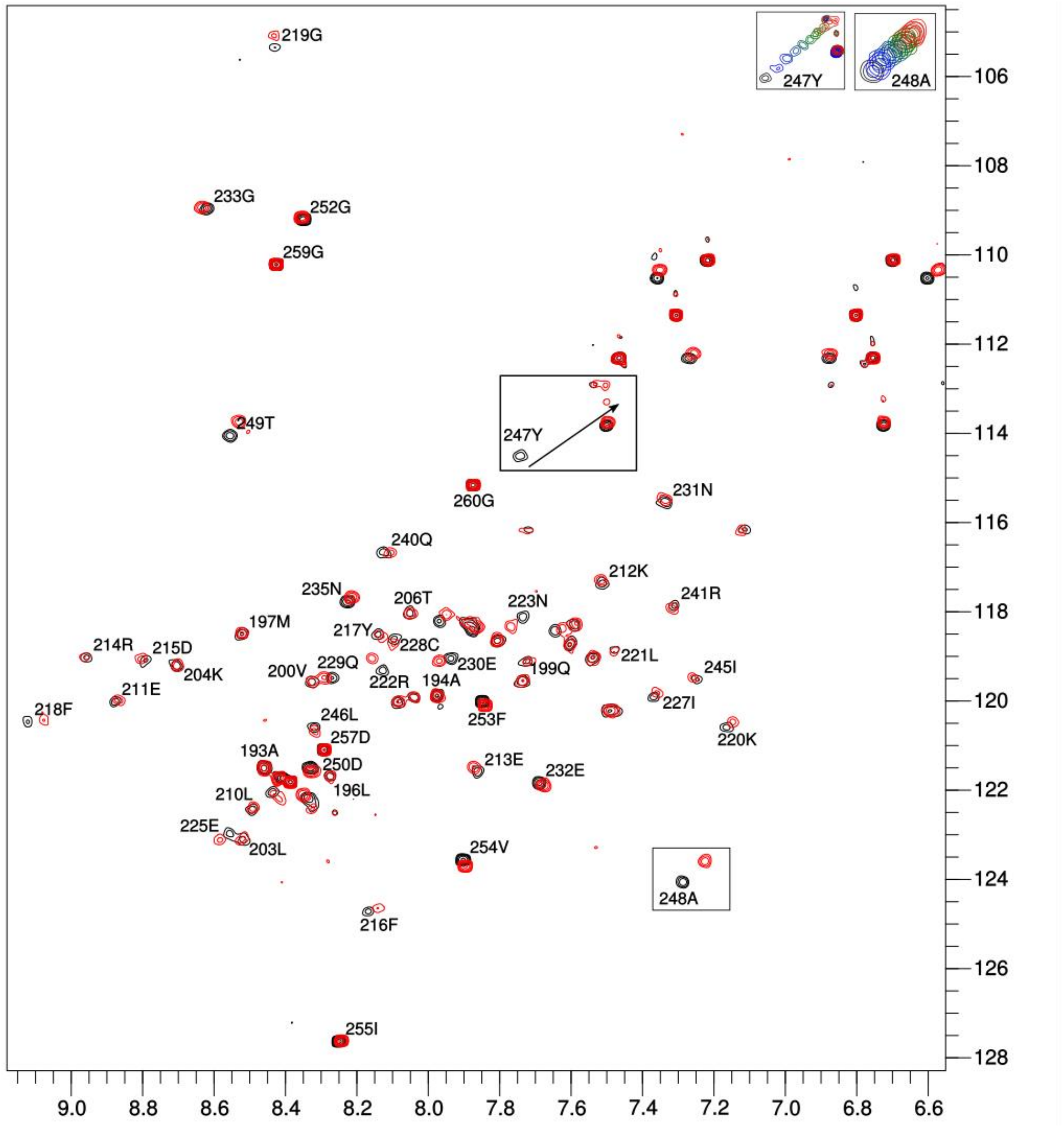

Figure S5 - Overlay of 2D ^{1}H-^{15}N – HSQC spectra recorded at 600 MHz – in black is presented EB1cΔ8 in the free form (50 µM) and in red the complex EB1cΔ8 (50 µM) – **1d** (5000 µM). The insets show superposition of signals of the most affected residues Tyr247 and Ala248 (highlighted by boxes) at the **1d** concentrations of 500, 1000, 1500, 2000, 2500, 3000, 3500, 4000, 4500 and 5000 µM used in the titration.

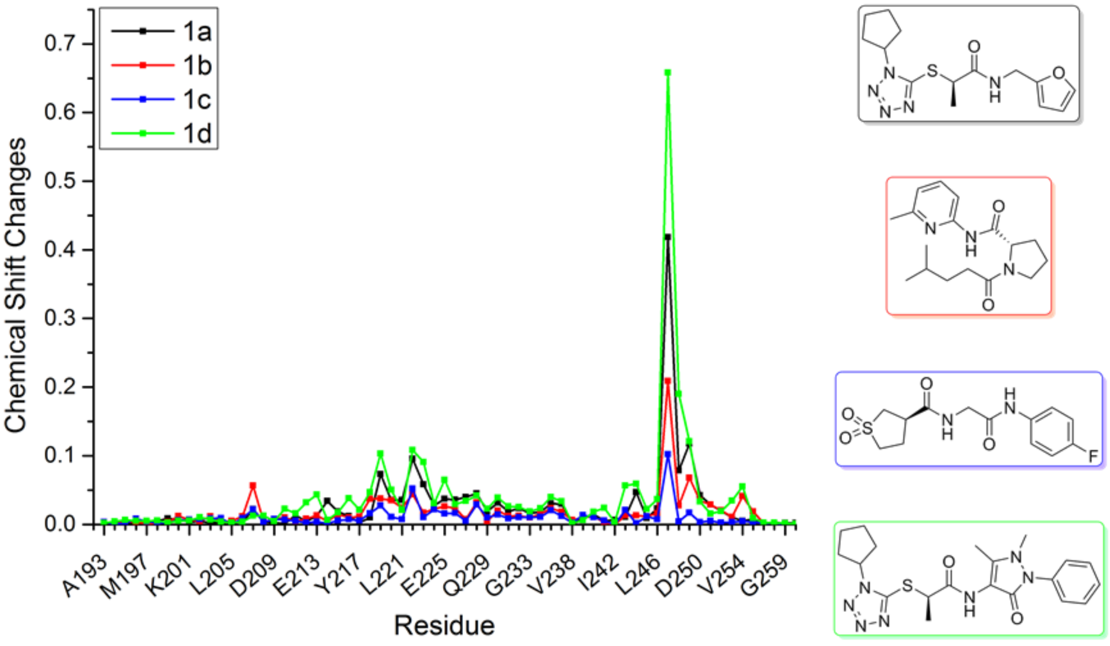

Figure S6 – Chemical shift changes plot for the four tested compounds and their distribution *per* EB1c residues. **1a** (black) and **1d** (green) seem to promote significant chemical shift changes in the resonances of the EB1c backbone, especially in two regions, Lys220-Glu225 and Leu246-Thr249.

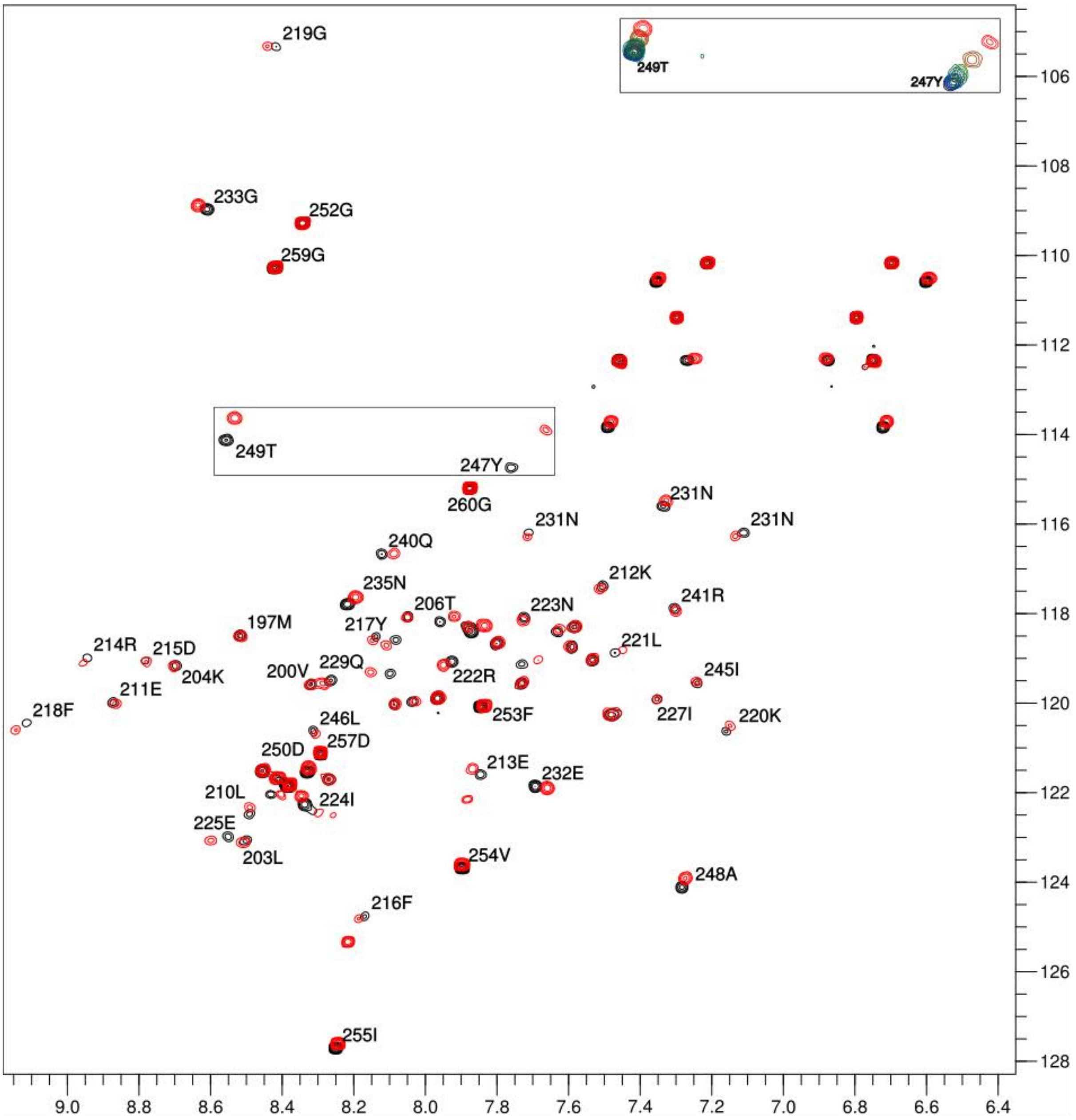

Figure S7 - Overlay of 2D ^{1}H-^{15}N – HSQC spectra recorded at 600 MHz – in black is presented EB1cΔ8 in the free form (50 µM) and in red the complex EB1cΔ8 (50 µM) – SKIP (5000 µM). The insets show superposition of signals of the most affected residues Tyr247 and Thr249 (highlighted by boxes) at the ligand concentrations of 250, 500, 1000, 2500 and 5000 µM used in the titration.

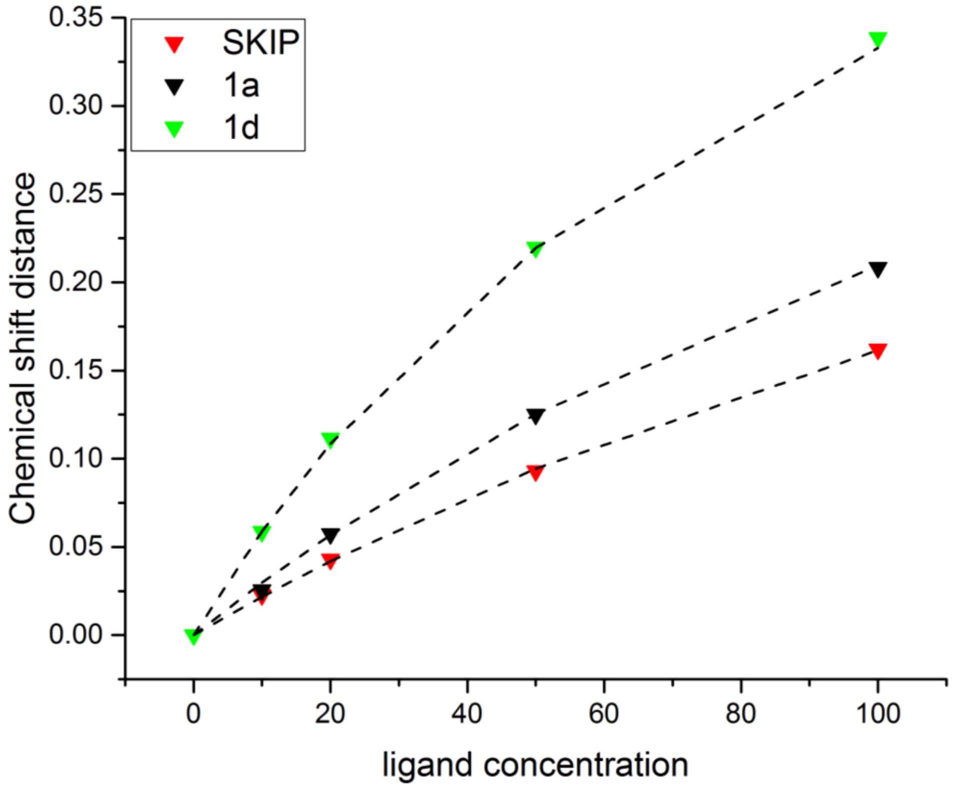

Figure S8 – Binding curves for residue Tyr247 for the titrations of the tetramer SKIP (red), compound **1a** (black) and compound **1d** (green). The dotted lines connect the values calculated for the best-fit curves at the titration points. Ligand concentration corresponds to the excess of ligand (*e.g.* 10-fold excess, 20-fold excess) to the protein concentration (50 μM). Binding constants were evaluated by fitting the experimental data to the equation

$$\Delta\delta_{obs} = \Delta\delta_{max}\left\{([P]_t + [L]_t + K_d) - [([P]_t + [L]_t + K_d)^2 - 4[P]_t [L]_t]^{1/2}\right\}/2[P]_t$$

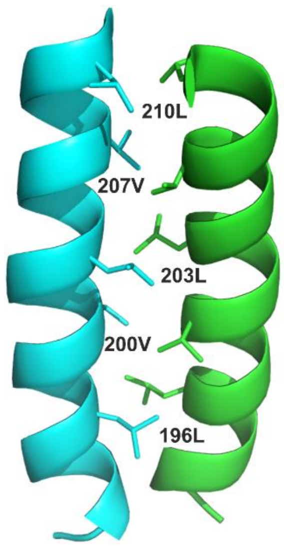

Figure S9 - Heptad repeat for the coiled coil structure of EB1c, side chains forming the apolar contacts are shown as sticks.

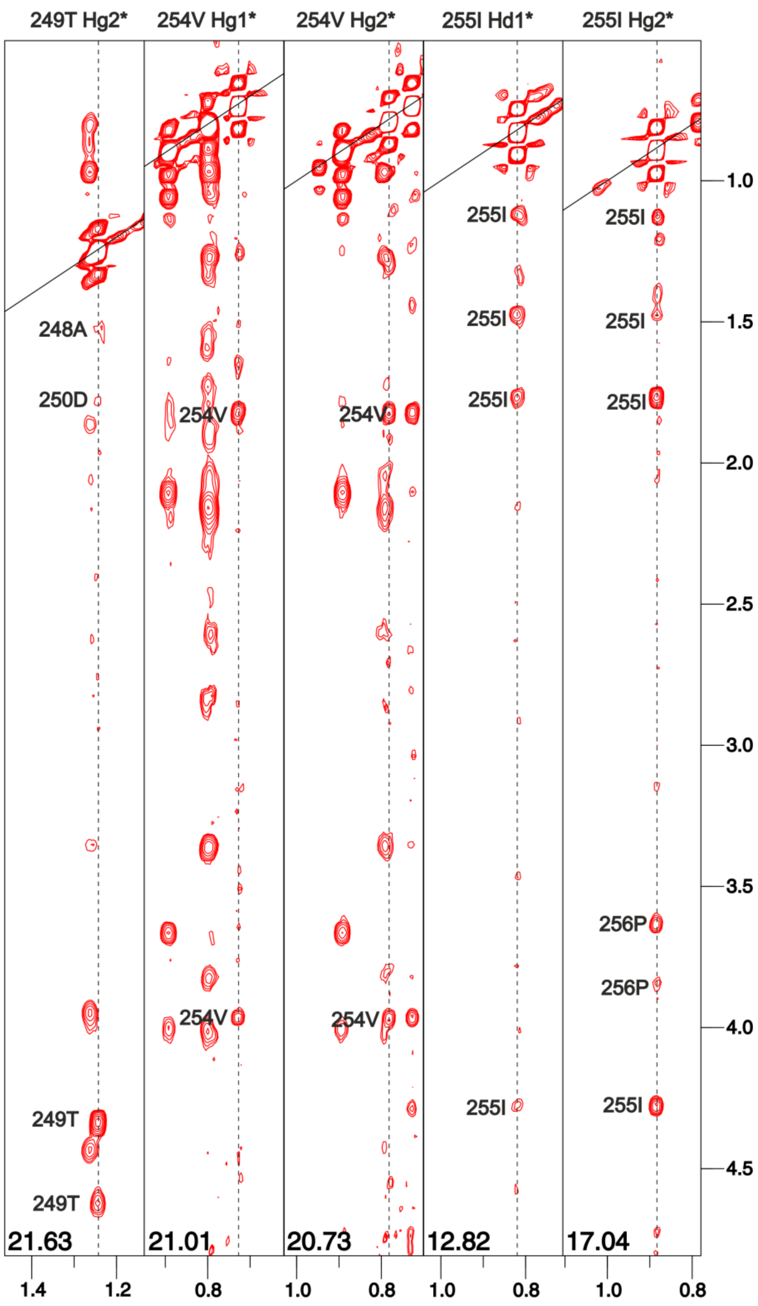

Figure S10 - Strips for the ^{13}C-resolved-NOESY-HSQC for the methyl groups of the following residues of the C-terminus of EB1c in the free form – Thr249, Val254 and Ile255. The contacts shown refer to the aliphatic region ~4.5 ppm to ~0.4 ppm and it is possible to observe the absence of inter-residue contacts, except for some sequential residues.

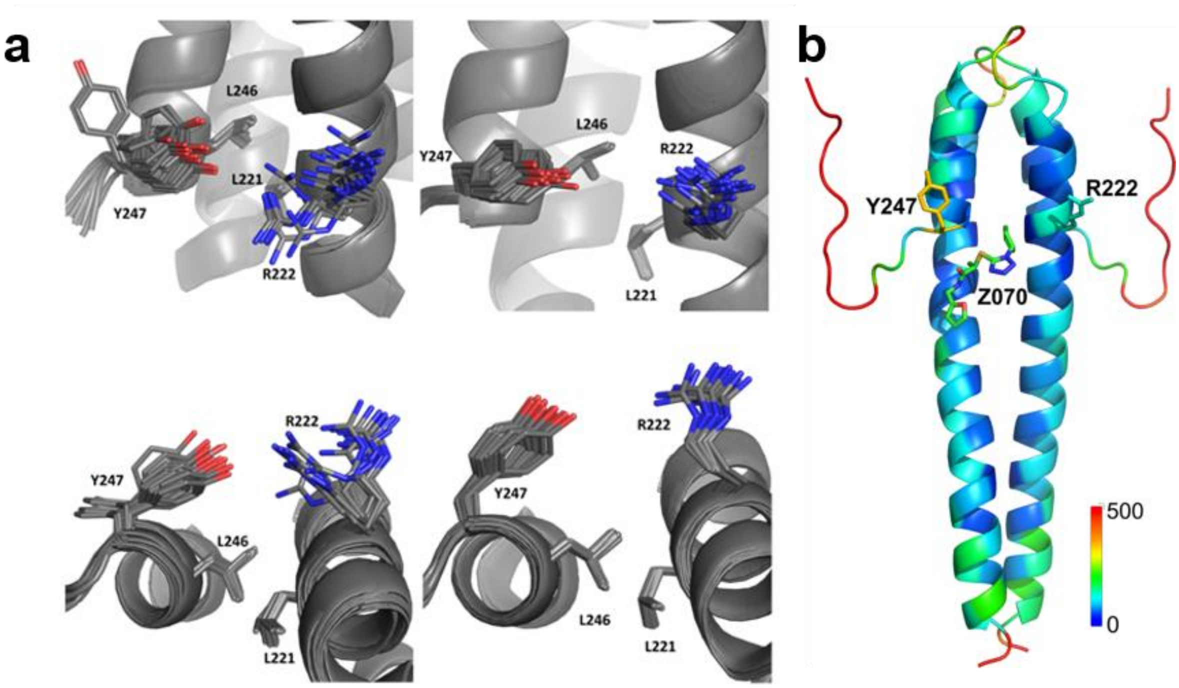

Figure S11 – a – conformations for Arg222 and Tyr247 for all the 20 structures calculated for free EB1 (left hand side) and bound to compound **1a** (right hand side). b – Lowest energy structure of free EB1s illustrating residue-specific root mean square fluctuations (RMSF) calculated from the 50 ns trajectory starting from the open orientation of Tyr247 side-chain.

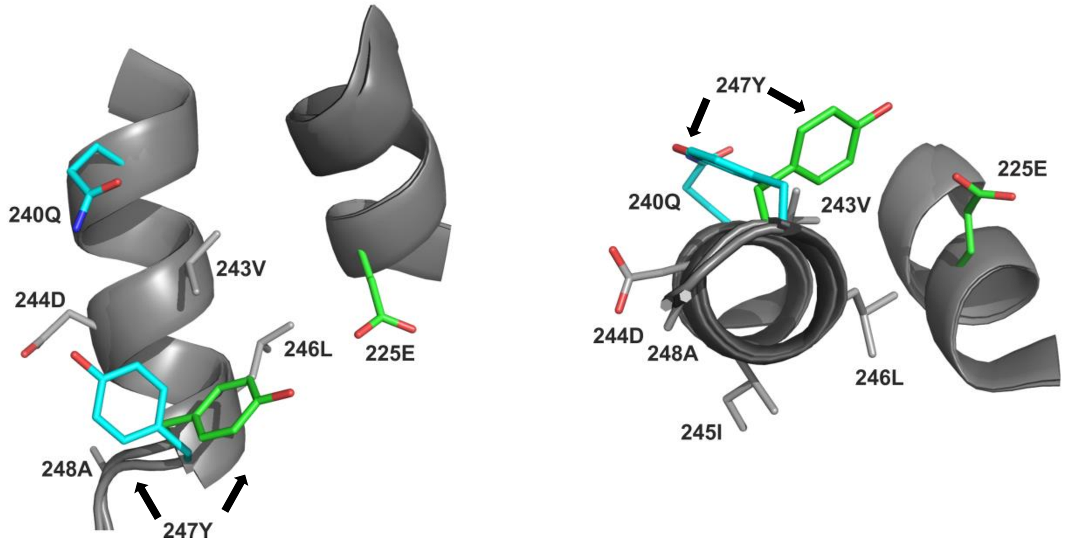

Figure S12 - Superimposition of the 2 lowest energy structures of the ensemble obtained for EB1c in the free form. In green it can be seen that Tyr247 is closer to Glu225, whereas in cyan it is closer to Gln240. The remaining residues that show distance restraints to Tyr247 are represented in grey sticks.

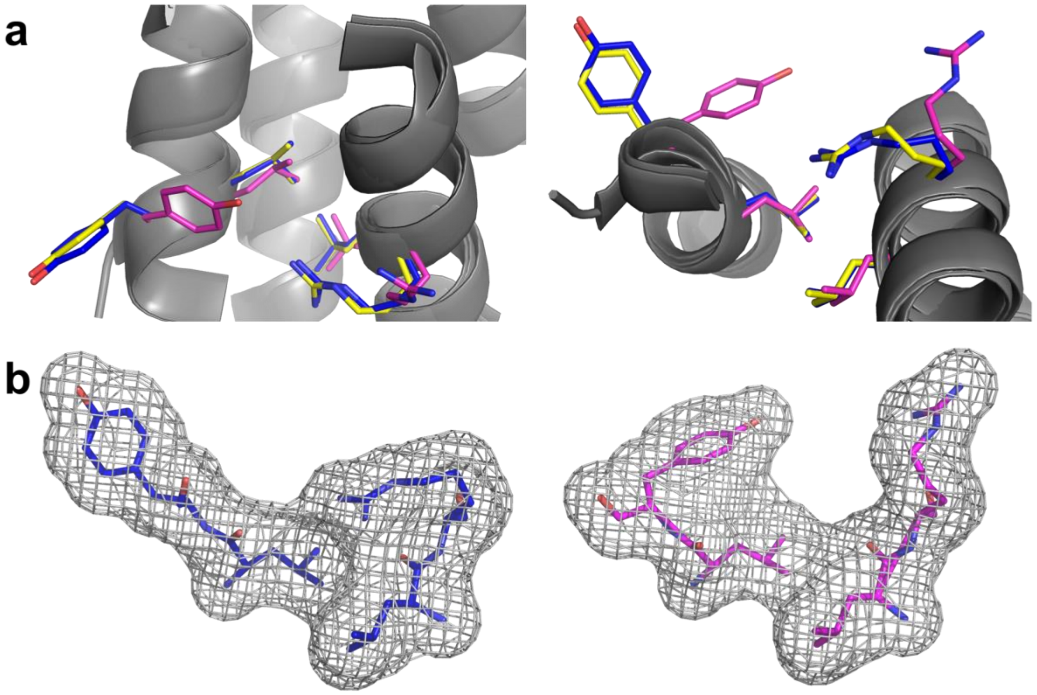

Figure S13 – a - Two conformations for residues Arg222 and Tyr247 in free (yellow and navy, PDB codes 1YIG and 1WU9 respectively) and bound state (magenta, PDB code 3GJO). Leu221 and Leu246 remain in a stable conformation as they are part of the coiled coil hydrophobic interface and are shown as reference points. On the right hand side the same representation with a 270° rotation on the *x* axis. b – Representation of the EB1 pocket shape based on the conformation of residues Arg222 and Tyr247, for unbound EB1c (PDB code 1YIG), and bound EB1c (PDB code 3GJO).

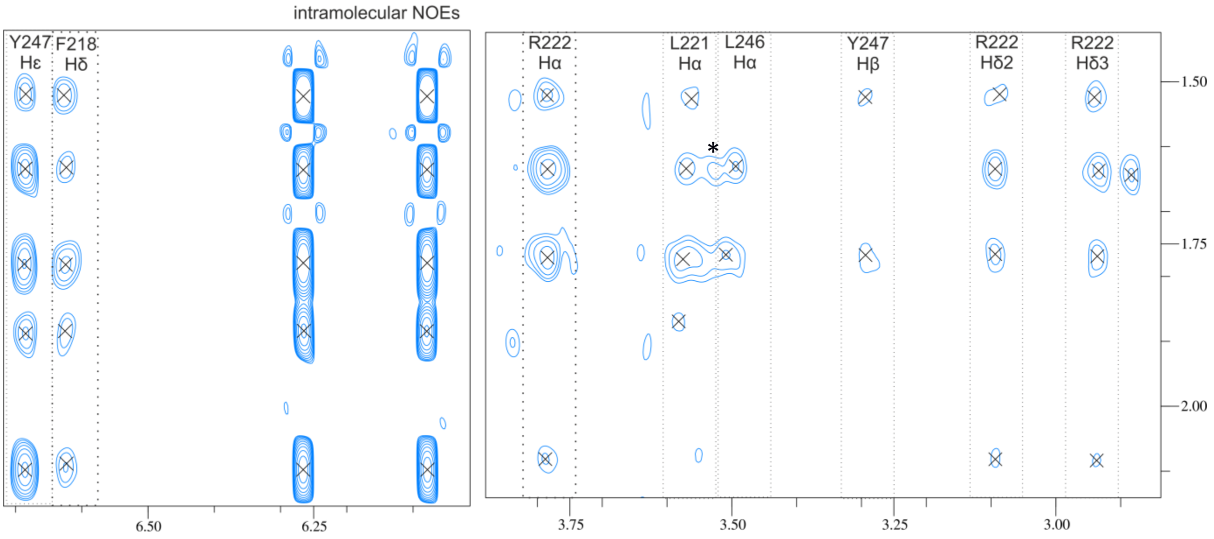

Figure S14 - Selected regions of a $^{13}C,^{15}N$ filtered-2D-NOESY (mixing time 200 ms) showing intra and intermolecular NOEs observed for EB1c-**1a** complex, at 25°C. The intramolecular NOEs correspond to the aromatic protons of the oxazole moiety NOEs to the cyclopentyl ring. The intermolecular NOEs show the NOE contacts between the cyclopentyl ring and Phe218, Leu221, Arg222, Leu246 and Tyr247, all part of the SxIP recruiting region. Cross-peak marked by "*" corresponds to NOE between the ligand and H$^{\alpha}$ of Glu225. This NOE was not used in the calculation as it was not reliably assigned due to the absence of supporting NOEs.

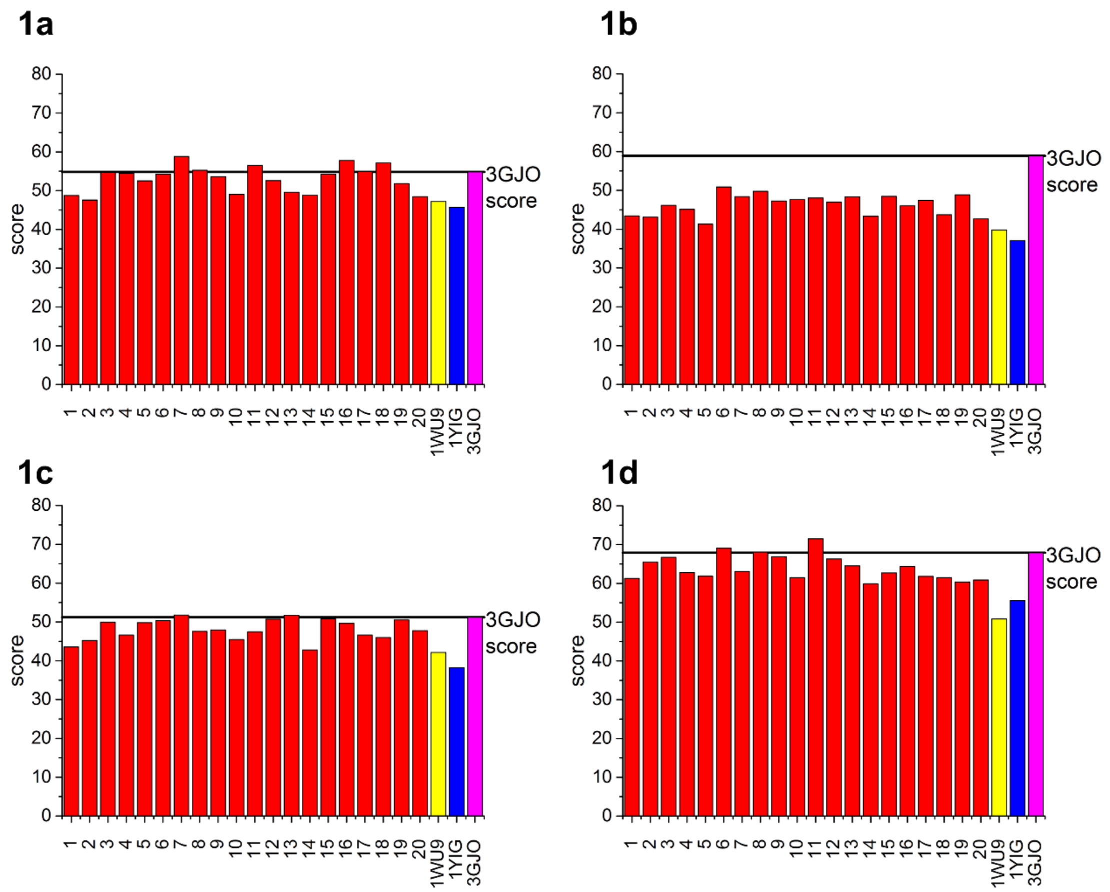

Figure S15 - predicted docking scores for the ensemble of the solution NMR structures of free EB1 (red), crystal structures of free EB1, 1WU9 (yellow) and 1YIG (navy), and crystal structure of EB1 in the bound state with SxIP protein, 3GJO (magenta). The score obtained for the reference structure where the initial docking calculations were performed – 3GJO, is defined by a black horizontal line to facilitate the comparison.

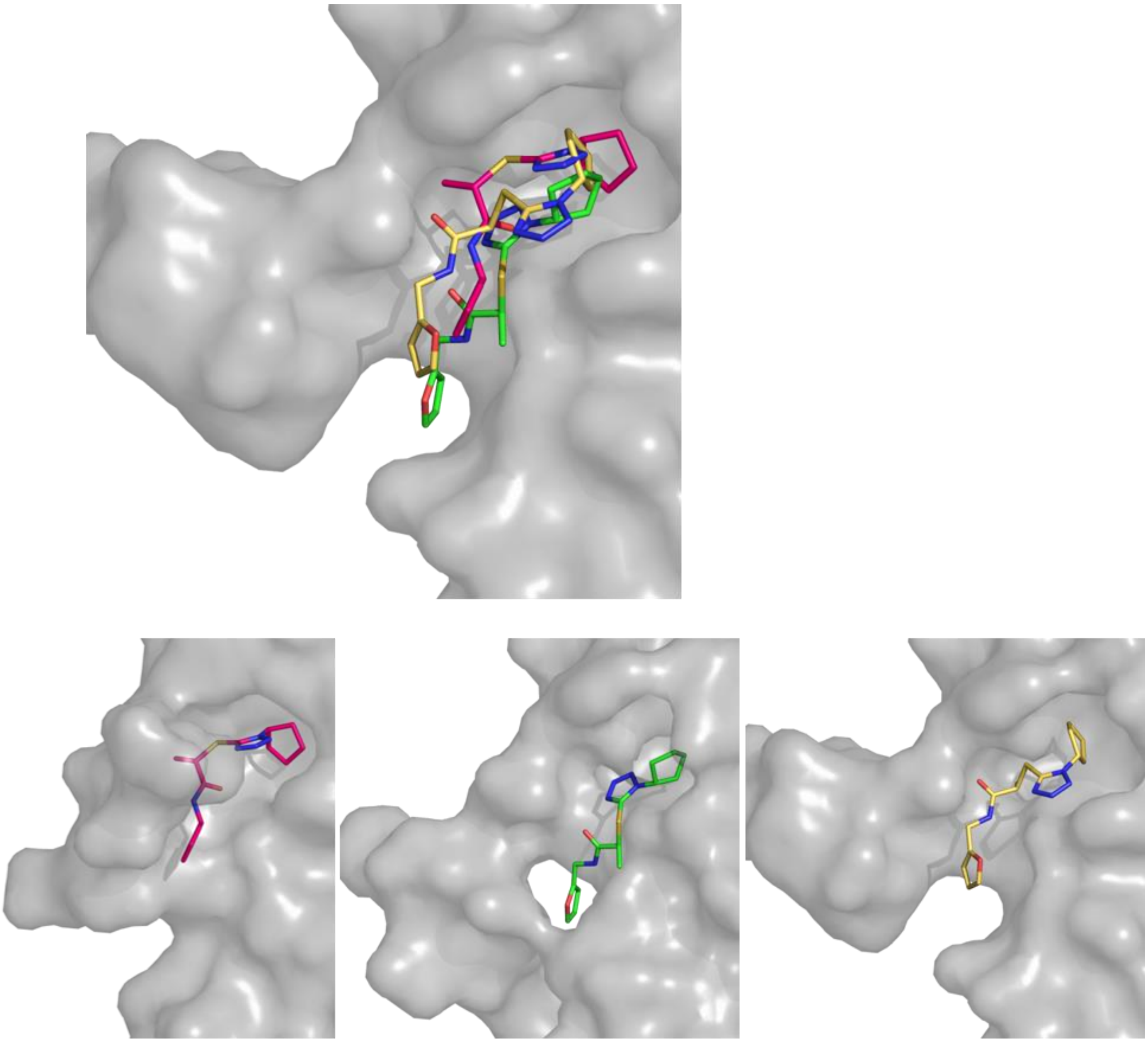

Figure S16 – Top panel - models obtained for compound **1a** superimposed on the same model of EB1Δc8 (corresponds to one of the NMR models obtained for free EB1). Lower panel, left - best scored docking pose obtained for the initial docking studies of compound **1a** and EB1 (PDB code 3GJO); middle – average of the NMR ensemble obtained for the structure of the complex between EB1Δc8 and **1a**; right – best scored docking pose obtained for compound **1a** on the NMR ensemble of free EB1Δc8.

Table S1- NMR restraints and structure statistics for the structures of free EB1c and in complex with molecule

	EB1c	EB1c-1a
**Total restraints used**		
NOE restraints*		
All	2641	2766
Protein-ligand	NA	75
Intermonomer	634 (99)	648 (99)
Intraresidue	980	1044
Sequential ($\|i-j\| = 1$)	633	641
Medium ($1 < \|i-j\| \leq 4$)	850	866
Long range ($\|i-j\| > 4$)	169	169
Dihedral		
ϕ *angles*	64	64
φ *angles*	64	64
Hydrogen bonds	90	90
**Structure statistics**		
Violations		
Distance (> 0.5 Å)	3	16
Dihedral angle ($> 5^0$)	1	0
Energies (cal/mol)		
Overall	-5688 (±169)	-5716 (±163)
Bond	26 (±2)	38 (±1.5)
Angle	150 (±7)	167 (±5.5)
Improper	307 (±50)	298 (±32)
Dihedral	697 (±9)	713 (±7)
Van der Waals	-1293 (±12)	-1298 (±10)
Electrostatic	-5576 (±158)	-5635 (±17)
NOE	352 (±45)	1280 (±50)
Geometry – average Values		
Bond	3.45×10^{-3} ($\pm 1.14 \times 10^{-4}$)	4.65×10^{-3} ($\pm 1.18 \times 10^{-4}$)
Angle	0.49 (±0.018)	0.67 (±0.013)
Improper	1.34 (±0.11)	1.31 (±0.071)
Dihedral	40.83 (±0.25)	40.94 (±0.25)
Van der Waals	162.96 (±12.05)	346.16 (±23.53)
Average pairwise RMSD (Å)**		
Heavy atoms	5.36 (±1.13)	4.54 ($\pm 9.28 \times 10^{-1}$)
Heavy atoms – helical region	0.89 (±0.09)	6.12×10^{-1} ($\pm 7.73 \times 10^{-2}$)
Backbone	5.75 (±1.23)	4.92 (±1.12)
Backbone – helical region	0.32 (±0.07)	1.57×10^{-1} ($\pm 3.61 \times 10^{-2}$)
Ramachandran statistics (%)		
Most favoured regions	90.8 (98.8)	90.6 (98.1)
Additional allowed regions	8.8 (1.2)	9.3 (1.9)
Generously allowed regions	0.4 (0)	0.1 (0)
Disallowed regions	0.0 (0)	0.0 (0)
